# Changes in blood metabolomes as potential markers for severity and prognosis in doxorubicin-induced cardiotoxicity: a study in HER2-positive and HER2-negative breast cancer patients

**DOI:** 10.1186/s12967-024-05088-9

**Published:** 2024-04-29

**Authors:** Chanisa Thonusin, Nichanan Osataphan, Krit Leemasawat, Wichwara Nawara, Sirawit Sriwichaiin, Siriporn Supakham, Siriluck Gunaparn, Nattayaporn Apaijai, Areewan Somwangprasert, Arintaya Phrommintikul, Siriporn C. Chattipakorn, Nipon Chattipakorn

**Affiliations:** 1 Cardiac Electrophysiology Unit, Department of Physiology, Faculty of Medicine, Chiang Mai University, Chiang Mai, Thailand; 2 Cardiac Electrophysiology Research and Training Center, Faculty of Medicine, Chiang Mai University, Chiang Mai, Thailand; 3 Center of Excellence in Cardiac Electrophysiology Research, Chiang Mai University, Chiang Mai, Thailand; 4 Division of Cardiology, Department of Internal Medicine, Faculty of Medicine, Chiang Mai University, Chiang Mai, Thailand; 5 Department of Surgery, Faculty of Medicine, Chiang Mai University, Chiang Mai, Thailand; 6 Department of Oral Biology and Diagnostic Sciences, Faculty of Dentistry, Chiang Mai University, Chiang Mai, Thailand

**Keywords:** Doxorubicin, Cardiotoxicity, Heart failure, HER2, Breast cancer, Metabolomes

## Abstract

**Background:**

We aimed to compare the changes in blood metabolomes and cardiac parameters following doxorubicin treatment in HER2-positive and HER2-negative breast cancer patients. Additionally, the potential roles of changes in blood metabolomes as severity and prognostic markers of doxorubicin-induced cardiotoxicity were determined.

**Methods:**

HER2-positive (n = 37) and HER2-negative (n = 37) breast cancer patients were enrolled. Cardiac function assessment and blood collection were performed at baseline and 2 weeks after completion of doxorubicin treatment in all patients, as well as at three months after completion of doxorubicin treatment in HER2-negative breast cancer patients. Blood obtained at all three-time points was processed for measuring cardiac injury biomarkers. Blood obtained at baseline and 2 weeks after completion of doxorubicin treatment were also processed for measuring systemic oxidative stress and 85 metabolome levels.

**Results:**

Cardiac injury and systolic dysfunction 2 weeks after completion of doxorubicin treatment were comparable between these two groups of patients. However, only HER2-negative breast cancer patients exhibited increased systemic oxidative stress and cardiac autonomic dysfunction at this time point. Moreover, 33 and 29 blood metabolomes were altered at 2 weeks after completion of doxorubicin treatment in HER2-positive and HER2-negative breast cancer patients, respectively. The changes in most of these metabolomes were correlated with the changes in cardiac parameters, both at 2 weeks and 3 months after completion of doxorubicin treatment.

**Conclusions:**

The changes in blood metabolomes following doxorubicin treatment were dependent on HER2 status, and these changes might serve as severity and prognostic markers of doxorubicin-induced cardiotoxicity.

*Trial registration*: The study was conducted under ethical approval from the Institutional Review Board of the Faculty of Medicine, Chiang Mai University (Registration number: MED-2563-07001; Date: April 28, 2020). The study also complied with the Declaration of Helsinki.

**Supplementary Information:**

The online version contains supplementary material available at 10.1186/s12967-024-05088-9.

## Introduction

Breast cancer is the most commonly diagnosed cancer among women [1, 2]. In 2020, there were approximately 2.3 million cases of breast cancer, with expectations to reach 4.4 million cases in 2070 [1, 2]. The increased incidence of breast cancer is mainly due to the increased prevalence of breast cancer risk factors such as obesity, physical inactivity, early menarche, shorter breastfeeding periods, and the use of oral contraceptive pills [3]. Nonetheless, the mortality rate of breast cancer has gradually declined in the past 30 years [4]. This decreasing trend is owing to the ability of early detection and the more effectiveness of therapeutic paradigms for breast cancer [4].

According to the National Comprehensive Cancer Network (NCCN)’s clinical guidelines, surgery is the primary treatment for breast cancer [5]. In addition to surgery, chemotherapy is considered an adjuvant therapy and a neoadjuvant therapy for early-stage breast cancer and locally advanced-stage breast cancer, respectively [5]. Importantly, chemotherapy can be the first-line therapeutic regimen for metastatic breast cancer [5]. Anthracyclines have been recognized as a standard component of chemotherapy for breast cancer since they effectively improve disease-free survival and overall survival when compared to other chemotherapeutic agents [6, 7]. Unfortunately, the use of anthracyclines is often limited due to their adverse effects, especially cardiotoxicity [8]. Currently, dexrazoxane is the only approved medication to protect against anthracycline-induced heart failure [9]. However, dexrazoxane exerts a myelosuppressive effect and can cause secondary malignant neoplasm [10, 11]. Therefore, the establishment of safer interventions for the prevention and treatment of anthracycline-induced heart failure is required.

Doxorubicin is an anthracycline that is commonly used as a chemotherapy for breast cancer [12]. Our prior study demonstrated that doxorubicin led to abnormal cardiac metabolism, as indicated by the alterations of cardiac metabolome levels in doxorubicin-treated rats [13]. Interestingly, these changes were robustly associated with the development of doxorubicin-induced heart failure [13]. All of these results supported the fact that cardiac metabolic impairment is a crucial component of heart failure [14]. Indeed, it is likely that cardiac metabolism plays an important role in determining the severity of heart failure induced by doxorubicin. It is well known that metabolomics in cardiac tissue is an analytical profiling technique that provides an in-depth understanding of cardiac metabolism, both in physiological and pathological conditions [15]. Unfortunately, measurements of cardiac metabolome levels are rarely possible in humans. Therefore, non-invasive biomarkers as representatives of cardiac metabolism are required in clinical research and practice. We previously revealed significant correlations between blood metabolome levels versus cardiac metabolome levels and cardiac functions in doxorubicin-induced heart failure rats, suggesting that blood metabolomes could be used as severity markers for doxorubicin-induced heart failure [16]. Nevertheless, whether blood metabolomes can be used as prognostic markers for doxorubicin-induced heart failure has never been investigated.

Besides doxorubicin-induced cardiotoxicity, human epidermal growth factor receptor 2 (HER2) is also a point of interest in breast cancer treatment. HER2-positive breast cancer approximately accounts for 20–25% of all breast cancer patients [17]. Overexpression of HER2 is associated with more aggressiveness of tumors, leading to increased mortality in HER2-positive breast cancer patients [18]. For this reason, the adjuvant anti-HER2 treatment, such as trastuzumab, following doxorubicin treatment is strongly indicated in these patients [19]. However, it was widely observed that HER2 overexpression protected against systemic oxidative stress in rats and in breast cancer patients [20–22]. Additionally, it was discovered that HER2 maintained cardiac contractility, and hence protected against dilated cardiomyopathy [23–25]. According to these conflicting roles of HER2, we hypothesized that the alterations of blood metabolome levels and cardiac functions following doxorubicin treatment are different between HER2-positive and HER2-negative breast cancer patients. In this study, we compared the changes in blood metabolome levels, cardiac functions, and cardiac injury biomarkers at 2 weeks after completion of doxorubicin between HER2-positive and HER2-negative breast cancer patients. To identify potential severity markers of doxorubicin-induced heart failure, we also determined the relationships between the changes in blood metabolome levels and the changes in cardiac functions and cardiac injury biomarkers at 2 weeks after completion of doxorubicin. Furthermore, to identify potential prognostic markers of doxorubicin-induced heart failure, the correlations between the changes in blood metabolome levels at 2 weeks after completion of doxorubicin and the changes in cardiac functions and cardiac injury biomarkers at three months after completion of doxorubicin were also determined.

## Materials and methods

### Participants

Breast cancer patients who visited the Breast Surgery Clinic at Maharaj Nakorn Chiang Mai Hospital, Chiang Mai University, Chiang Mai, Thailand were enrolled in this study. The study was conducted under ethical approval by the Institutional Review Board (Study code: MED-2563-07001; Date: April 28, 2020) and was registered at the Thai Clinical Trials Registry (Identification number: TCTR20200116007). The study also complied with the Declaration of Helsinki. The informed consent process was done in all participants.

The inclusion criteria included (1) female patients ≥ 18 years of age, (2) newly diagnosed with breast cancer, (3) receiving surgical treatment, (4) plan to receive adjuvant chemotherapy (60 mg/m^2^ of doxorubicin plus 600 mg/m^2^ of cyclophosphamide intravenously every 21 days for four cycles), and (5) given the informed consent. The exclusion criteria were (1) metastatic breast cancer at diagnosis, (2) other active malignancies, (3) prior history of chemotherapy or chest wall radiation, (4) prior cardiovascular disease with left ventricular ejection fraction (LVEF) < 53% or severe valvular heart disease, (5) creatinine clearance < 30 mL/min/1.73 m^2^, (6) baseline electrocardiogram (ECG) showed corrected QT interval ≥ 500 ms, (7) life expectancy < 1 year, and (8) pregnancy or breastfeeding.

### Study protocol

The study protocol is illustrated in Additional file 1: Fig. S1. After receiving surgical treatment, patients were categorized into two groups based on their breast cancer pathology, including (1) HER2 positive and (2) HER2 negative. History taking, physical examination, echocardiography, heart rate variability, and blood collection were performed on all participants before the beginning of adjuvant chemotherapy (baseline). Echocardiography, heart rate variability, and blood collection were also done in all patients 2 weeks after completion of the adjuvant chemotherapy, and only in HER2-negative breast cancer patients three months after completion of the adjuvant chemotherapy. In other words, HER2-positive breast cancer patients were excluded three months after completion of the adjuvant chemotherapy because they already received trastuzumab treatment which might confound the findings. Blood obtained at baseline and 2 weeks after completion of adjuvant chemotherapy was processed for the measurement of cardiac injury biomarkers, systemic oxidative stress, and metabolomics study. Blood obtained three months after completion of adjuvant chemotherapy was processed for the measurement of cardiac injury biomarkers.

### Echocardiography

Complete 2-dimensional transthoracic echocardiography (Philips EPIQ CVx, Philips, Amsterdam, the Netherlands) under M mode and Doppler mode (pulsed wave, continuous wave, and color) was performed according to the recommendations of the American Society of Echocardiography [26]. LVEF was measured by modified biplane Simpson’s technique to determine systolic function. Diastolic function was determined by early mitral inflow velocity-to-mitral annular early diastolic velocity (E/e’) ratio.

### Heart rate variability

Holter (GE Seer Light Extend, GE Medical Systems, Suzuken Company, Limited, Aichi, Japan) monitoring was used for the measurement of heart rate variability. The recording duration was 10 min. Frequency-domain analysis was performed using autoregressive power spectral analysis applied to the RR interval time series according to the standard guidelines [27]. The parasympathetic tone was indicated by a high frequency (HF) in the range of 0.15–0.40 Hz. On the other hand, a low frequency (LF) in the range of 0.04–0.15 Hz represented both parasympathetic and sympathetic tones. A high LF/HF ratio indicated a cardiac sympathovagal imbalance, i.e., cardiac autonomic dysfunction [27].

### Cardiac injury biomarkers

Troponin I level in plasma was measured using chemiluminescent microparticle immunoassay (the Abbott/Architect stat high sensitivity Troponin I assay ARCHITECT i2000SR Diagnostic System) according to the manufacturer’s instruction (Abbott Laboratories, Lake Forest, IL, USA).

N-terminal pro-B-type natriuretic peptide (NT-proBNP) level was also determined in plasma using electrochemiluminescence immunoassay (Roche NT-proBNP assay) according to the manufacturer’s instruction (Roche Diagnostics, Basel, Switzerland).

### Isolation of peripheral blood mononuclear cells

Peripheral blood mononuclear cells were isolated from fresh blood using a Ficoll density gradient centrifugation as previously described [28]. Briefly, a ten-minute initial centrifugation at 1000 relative centrifugal force (RCF) was performed. Then, white and red blood cells were collected and re-suspended in a phosphate buffer saline solution. After that, white and red blood cells were over-layered on Ficoll-Paque reagent (Histopaque, Sigma-Aldrich, Saint Louis, MO, USA) and centrifuged at 400 RCF for 30 min. Subsequently, the ring of peripheral blood mononuclear cells at the Ficoll/plasma interface was collected, and then washed with phosphate buffer saline solution. The viability of peripheral blood mononuclear cells was stained with trypan blue dye, and cell number was counted by an automatic hemocytometer (NanoEntek, Waltham, MA, USA).

### Cellular oxidative stress in peripheral blood mononuclear cells

Cellular oxidative stress levels in peripheral blood mononuclear cells were measured to determine systemic oxidative stress. In detail, 2 × 10^5^ of peripheral blood mononuclear cells were stained with 2 μM of dichloro-dihydro-fluorescein diacetate (DCFH-DA) dye. The fluorescent intensity of dichlorofluorescein (DCF) indicated cellular oxidative stress level, which was detected by a flow cytometer (BD FACS Celesta, BD Biosciences, Franklin Lakes, NJ, USA).

### Plasma metabolome extraction

One hundred μL of an ice-cold extraction solvent (1:1:1 of methanol: acetonitrile: acetone) was added to 25 μL of plasma. The samples were vortexed for 15 s and then centrifuged at 16,800 revolutions per minute (rpm) for ten minutes at 4 °C. The supernatant was finally collected.

### Plasma metabolome levels

A targeted metabolomics approach using liquid chromatography coupled with quadrupole time-of-flight mass spectrometry (LC/Q-TOF MS) was conducted for the measurement of 85 plasma metabolome levels. These metabolomes are involved in metabolic pathways for fuel utilization of the heart, which play crucial roles in maintaining normal cardiac metabolism, including amino acids, free fatty acids, acylcarnitines, Krebs cycle metabolomes, lactate, and acetoacetate [29]. Because the plasma levels of most glycolysis metabolomes were lower than the limit of detection of LC/Q-TOF MS [30], these metabolomes were not included in our plasma metabolomics study. Phospholipids were also included as our targeted metabolomes since they are important structural and functional components of the myocardial cell membrane and cardiac mitochondrial membrane [31]. All 85 metabolomes are listed in Additional file 1: Table S1.

Regarding the liquid chromatography (LC) part, plasma metabolome levels were quantitated using a 1260 infinity II LC/6546 Q-TOF MS (Agilent Technologies, Santa Clara, CA, USA). The LC condition for each metabolome is shown in Additional file 1: Table S1. The protocols of all LC conditions, mass spectrometry parameters, and data normalization were described in previous studies [16, 32]. The injection volume was 5 μL.

### Statistical analysis

A chi-square test was used to compare the categorical variables between HER2-positive and HER2-negative breast cancer patients. For the numerical variables, values were screened for the normality by Kolmogorov–Smirnov Test using R (version 4.1.1). The results showed that all numerical variables were normally distributed, as indicated by p-values > 0.05. Then, Student’s t-test was applied. An unpaired two-tailed Student’s t-test was used to compare the numerical variables between these two groups of patients. A paired Student’s t-test was used to compare the numerical variables between two-time points of the same participant. For non-metabolomics data, a *p*-value of less than 0.05 was considered statistically significant. For metabolomics data, each *p*-value was adjusted by the Benjamini–Hochberg correction method using R (version 4.1.1) and MetaboAnalyst (version 5.0) [33]. A false discovery rate (FDR) value of less than 0.05 was considered statistically significant. Pearson’s correlation was also used to determine the relationships between the changes in plasma metabolome levels versus the changes in cardiac functions and cardiac injury biomarkers. A *p*-value of less than 0.05 was considered statistically significant.

## Results

### Baseline characteristics of patients

Thirty-seven HER2-positive (51.81 ± 1.73 years of age) and 37 age-matched HER2-negative (49.57 ± 1.81 years of age) breast cancer patients were included in this study. The baseline characteristics of each patient group are shown in Additional file 1: Table S2. Indeed, age, body mass index, body surface area, systolic blood pressure, diastolic blood pressure, and heart rate were not different between groups. Additionally, the number of patients with underlying diseases did not differ between groups.

### Cardiac functions, cardiac injury biomarkers, systemic oxidative stress, and plasma metabolome levels at baseline

**Fig. 1:**
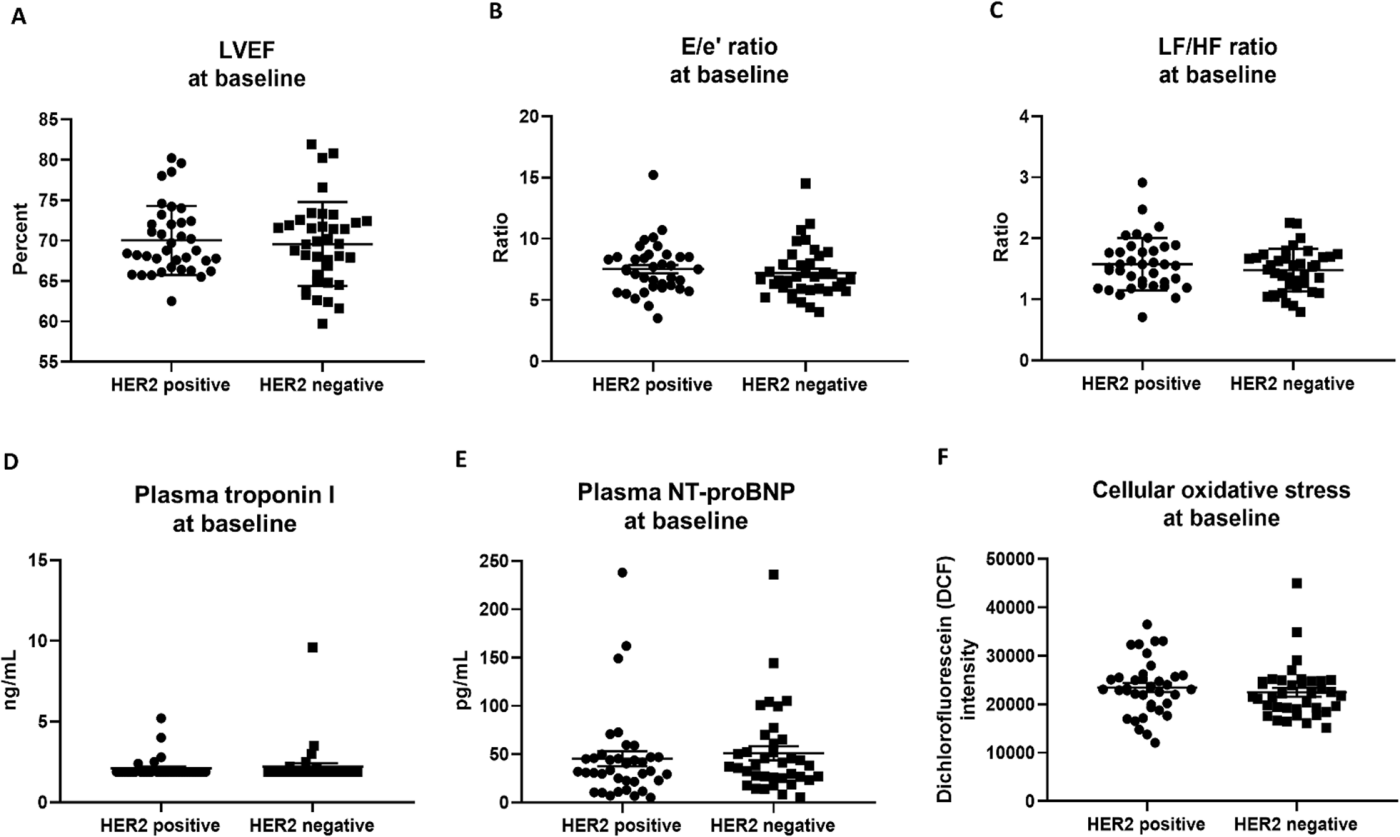
LVEF (**A**), E/e’ Ratio (**B**), LF/HF ratio (**C**), Plasma Troponin I (**D**), Plasma NT-proBNP (**E**), and Cellular Oxidative Stress in Peripheral Blood Mononuclear Cells (**F**) at Baseline in HER2-Positive Versus HER2-Negative Breast Cancer Patients. n = 37 per group. Data are reported as mean ± standard error of the mean (SEM). LVEF = Left ventricular ejection fraction; E/e’ ratio = Early mitral inflow velocity-to-mitral annular early diastolic velocity ratio; LF/HF ratio = Low frequency-to-high frequency ratio; NT-proBNP = N-terminal pro B-type natriuretic peptide; DCF = Dichlorofluorescein

Cardiac functions including LVEF, E/e’ ratio, and LF/HF ratio were evaluated before the beginning of adjuvant chemotherapy. The results showed that all of these parameters did not differ between HER2-positive and HER2-negative breast cancer patients (Fig. 1A–C). Cardiac injury biomarkers including plasma troponin I and NT-proBNP levels, and cellular oxidative stress in peripheral blood mononuclear cells were not different between the 2 groups of patients at this timepoint (Fig. 1D–F). Consistent with cardiac parameters and systemic oxidative stress, baseline plasma metabolome levels were not different between HER2-positive and HER2-negative breast cancer patients (see Additional file 1: Table S3). All of these findings suggested that HER2 status neither determined baseline cardiac parameters, baseline systemic oxidative stress, nor baseline blood metabolome levels.

### Cardiac functions, cardiac injury biomarkers, and systemic oxidative stress 2 weeks after completion of doxorubicin treatment

**Fig. 2:**
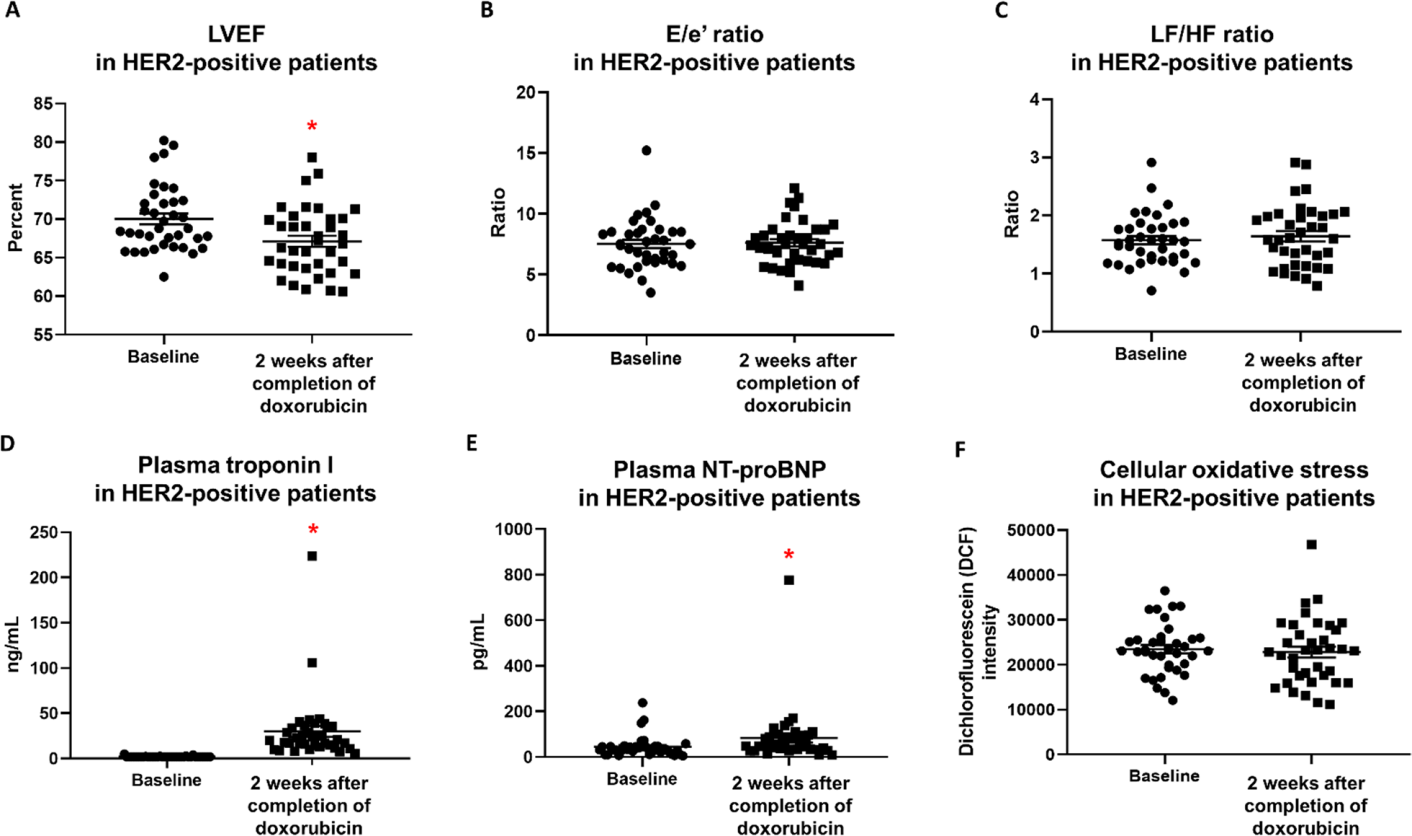
LVEF (**A**), E/e’ Ratio (**B**), LF/HF ratio (**C**), Plasma Troponin I (**D**), Plasma NT-proBNP (**E**), and Cellular Oxidative Stress in Peripheral Blood Mononuclear Cells (**F**) in HER2-Positive Breast Cancer Patients At Baseline Versus 2 Weeks After Completion of Doxorubicin Treatment. n = 37. Data are reported as mean ± standard error of the mean (SEM). *p < 0.05 when compared to baseline. LVEF = Left ventricular ejection fraction; E/e’ ratio = Early mitral inflow velocity-to-mitral annular early diastolic velocity ratio; LF/HF ratio = Low frequency-to-high frequency ratio; NT-proBNP = N-terminal pro B-type natriuretic peptide; DCF = Dichlorofluorescein

In HER2-positive breast cancer patients, LVEF was significantly decreased 2 weeks after completion of doxorubicin treatment (Fig. 2A), while the E/e’ ratio and LF/HF ratio were unchanged (Fig. 2B, C). Regarding cardiac injury biomarkers, plasma troponin I and NT-proBNP levels of HER2-positive breast cancer patients were significantly increased 2 weeks after completion of doxorubicin treatment (Fig. 2D, E). However, cellular oxidative stress in peripheral blood mononuclear cells of these patients was not different between these two-time points (Fig. 2F).

**Fig. 3:**
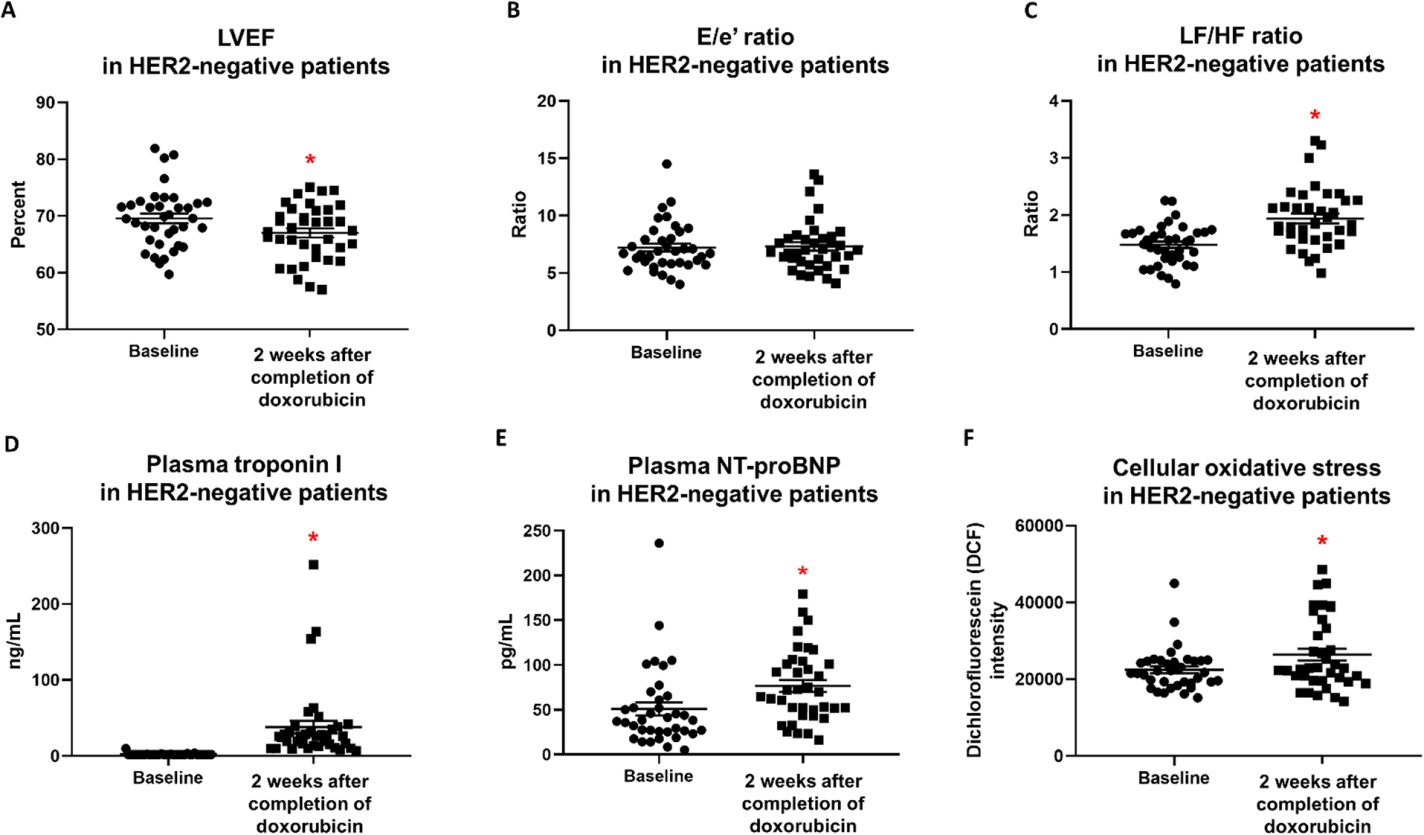
LVEF (**A**), E/e’ Ratio (**B**), LF/HF ratio (**C**), Plasma Troponin I (**D**), Plasma NT-proBNP (**E**), and Cellular Oxidative Stress in Peripheral Blood Mononuclear Cells (**F**) in HER2-Negative Breast Cancer Patients at Baseline Versus 2 Weeks After Completion of Doxorubicin Treatment. n = 37. Data are reported as mean ± standard error of the mean (SEM). * *p* < 0.05 when compared to baseline. LVEF = Left ventricular ejection fraction; E/e’ ratio = Early mitral inflow velocity-to-mitral annular early diastolic velocity ratio; LF/HF ratio = Low frequency-to-high frequency ratio; NT-proBNP = N-terminal pro B-type natriuretic peptide; DCF = Dichlorofluorescein

In HER2-negative breast cancer patients, LVEF was significantly decreased 2 weeks after completion of doxorubicin treatment (Fig. 3A), whereas E/e’ ratio was unaltered (Fig. 3B). Unlike their HER2-positive counterparts, LF/HF ratio and cellular oxidative stress in peripheral blood mononuclear cells of HER2-negative cancer patients were significantly increased 2 weeks after completion of doxorubicin treatment (Fig. 3C, F). Plasma troponin I and NT-proBNP levels were also significantly increased in these patients at this time point (Fig. 3D, E).

To determine whether the degree of changes in cardiac parameters and systemic oxidative stress 2 weeks after completion of doxorubicin treatment was different between HER2-positive and HER2-negative breast cancer patients, the absolute changes in these parameters were compared between these two groups of patients. Our results showed that the degree of changes in LVEF and cardiac injury biomarkers were not different between groups, while the increases in LF/HF ratio and cellular oxidative stress in peripheral blood mononuclear cells were significantly greater in HER2-negative than that of HER2-positive breast cancer patients (Additional file 1: Fig. S2). All of these findings indicated that cardiac systolic dysfunction and cardiac injury induced by doxorubicin were not dependent on HER2 status. However, HER2-negative breast cancer patients were more susceptible than HER2-positive breast cancer patients in the development of doxorubicin-induced cardiac autonomic dysfunction and systemic oxidative stress.

### Plasma metabolome levels 2 weeks after completion of doxorubicin treatment

In HER2-positive breast cancer patients, 33 of 85 plasma metabolome levels were significantly altered 2 weeks after completion of doxorubicin treatment, when compared to those of baseline (Table 1; Additional file 1: Table S4). These changes were involved in the metabolism of several amino acids, including glycine, phenylalanine, and branched-chain amino acids (Table 1; Additional file 1: Table S4). In addition, the levels of plasma arachidonic acid and several fatty acid-derived acylcarnitines in these patients were significantly decreased 2 weeks after completion of doxorubicin treatment (Table 1; Additional file 1: Table S4), suggesting doxorubicin-induced abnormal fatty acid metabolism. Several plasma phospholipid levels in these patients were also significantly changed 2 weeks after completion of doxorubicin treatment (Table 1; Additional file 1: Table S4), indicating the alterations of phospholipid metabolism induced by doxorubicin.

**Table 1:** List of 33 plasma metabolomes in HER2-positive breast cancer patients that were significantly altered (FDR < 0.05) at 2 weeks after completion of doxorubicin treatment

Significantly altered metabolomes (Total 33 metabolomes)	Fold change when compared to baseline
• Amino acids	
*Glycine*	*0.82* ± *0.07*
**Isoleucine and Leucine**	**1.19 ± 0.05**
**Phenylalanine**	**1.12 ± 0.04**
• Branched-chain amino acid-derived acylcarnitines	
*Propionylcarnitine*	*0.87* ± *0.05*
*Isobutyrylcarnitine*	*0.70* ± *0.04*
• Free fatty acids	
*Arachidonic acid*	*0.92* ± *0.10*
• Fatty acid-derived acylcarnitines	
*Acetylcarnitine*	*0.69* ± *0.06*
*Hexanoylcarnitine*	*0.62* ± *0.07*
*Octanoylcarnitine*	*0.52* ± *0.09*
*Octenoylcarnitine*	*0.42* ± *0.03*
*Decanoylcarnitine*	*0.57* ± *0.12*
*Decenoylcarnitine*	*0.51* ± *0.05*
*Lauroylcarnitine*	*0.70* ± *0.11*
*Dodecenoylcarnitine*	*0.64* ± *0.11*
*Myristoylcarnitine*	*0.87* ± *0.04*
*Tetradecenoylcarnitine*	*0.77* ± *0.11*
*Tetradecadienoylcarnitine*	*0.67* ± *0.09*
*Palmitoylcarnitine*	*0.88* ± *0.05*
*Palmitoleoylcarnitine*	*0.77* ± *0.08*
*Hexadecadienoylcarnitine*	*0.77* ± *0.09*
*Oleylcarnitine*	*0.79* ± *0.06*
*Linoleylcarnitine*	*0.67* ± *0.04*
• Krebs’ cycle metabolomes	
*Malate*	*0.85* ± *0.05*
• Phospholipids	
*Lysophosphatidylcholine (16:0)*	*0.94* ± *0.02*
**Lysophosphatidylethanolamine (18:1)**	**1.79 ± 0.15**
**Lysophosphatidylinositol (18:1)**	**1.33 ± 0.07**
**Phosphatidic acid (34:1)**	**2.16 ± 0.39**
**Phosphatidylcholine (36:1)**	**1.15 ± 0.04**
**Phosphatidylcholine (36:2)**	**1.03 ± 0.02**
**Phosphatidylethanolamine (34:1)**	**1.20 ± 0.05**
**Phosphatidylethanolamine (38:4)**	**1.48 ± 0.21**
**Phosphatidylserine (38:4)**	**1.74 ± 0.20**
**Phosphatidylserine (40:6)**	**1.28 ± 0.11**

n = 37. Fold change of each metabolome was defined as a ratio of metabolome level at 2 weeks and the baseline metabolome level of each patient. Then, data are reported as mean ± standard error of the mean (SEM)

**Bold**: Significantly increased when compared to baseline

*Italic*: Significantly decreased when compared to baseline

In HER2-negative breast cancer patients, 29 of 85 plasma metabolome levels were significantly altered 2 weeks after completion of doxorubicin treatment, when compared to those of baseline (Table 2; Additional file 1: Table S5). These changes were involved in the metabolism of several amino acids, including glutamine, tryptophan, and branched-chain amino acids (Table 2; Additional file 1: Table S5). Consistent with HER2-positive breast cancer patients, the levels of plasma arachidonic acid and several fatty acid-derived acylcarnitines in HER2-negative breast cancer patients were significantly decreased 2 weeks after completion of doxorubicin treatment (Table 2; Additional file 1: Table S5). These indicated doxorubicin-induced abnormal fatty acid metabolism. Several plasma phospholipid levels in these patients were also significantly altered 2 weeks after completion of doxorubicin treatment (Table 2; Additional file 1: Table S5), suggesting the changes in phospholipid metabolism induced by doxorubicin.

**Table 2:** List of 29 plasma metabolomes in HER2-negative breast cancer patients that were significantly altered (FDR < 0.05) at 2 weeks after completion of doxorubicin treatment

Significantly altered metabolomes (Total 29 metabolomes)	Fold change when compared to baseline
• Amino acids	
**Glutamine**	**1.15 ± 0.05**
*Tryptophan*	*0.82* ± *0.04*
• Branched-chain amino acid-derived acylcarnitines	
*Propionylcarnitine*	*0.87* ± *0.07*
*Isobutyrylcarnitine*	*0.74* ± *0.04*
*Isovalerylcarnitine*	*0.83* ± *0.08*
• Free fatty acids	
*Arachidonic acid*	*0.86* ± *0.10*
• Fatty acid-derived acylcarnitines	
*Acetylcarnitine*	*0.74* ± *0.06*
*Hexanoylcarnitine*	*0.83* ± *0.10*
*Octanoylcarnitine*	*0.57* ± *0.11*
*Octenoylcarnitine*	*0.61* ± *0.09*
*Decanoylcarnitine*	*0.55* ± *0.11*
*Decenoylcarnitine*	*0.57* ± *0.07*
*Lauroylcarnitine*	*0.67* ± *0.10*
*Dodecenoylcarnitine*	*0.62* ± *0.11*
*Myristoylcarnitine*	*0.90* ± *0.03*
*Tetradecenoylcarnitine*	*0.76* ± *0.12*
*Tetradecadienoylcarnitine*	*0.70* ± *0.11*
*Palmitoylcarnitine*	*0.88* ± *0.06*
*Palmitoleoylcarnitine*	*0.82* ± *0.08*
*Hexadecadienoylcarnitine*	*0.83* ± *0.10*
*Oleylcarnitine*	*0.86* ± *0.06*
*Linoleylcarnitine*	*0.86* ± *0.07*
• Phospholipids	
*Lysophosphatidylcholine (16:0)*	*0.93* ± *0.02*
**Lysophosphatidylethanolamine (18:1)**	**1.85 ± 0.17**
**Phosphatidylcholine (36:2)**	**1.07 ± 0.02**
*Phosphatidylglycerol (34:1)*	*0.88* ± *0.08*
*Phosphatidylglycerol (36:1)*	*0.90* ± *0.05*
*Phosphatidylglycerol (36:2)*	*0.92* ± *0.09*
**Phosphatidylserine (38:4)**	**1.75 ± 0.15**

n = 37. Fold change of each metabolome was defined as a ratio of metabolome level at 2 weeks and the baseline metabolome level of each patient. Then, data are reported as mean ± standard error of the mean (SEM)

**Bold**: Significantly increased when compared to baseline

*Italic*: Significantly decreased when compared to baseline

Considering only altered plasma metabolomes, 23 plasma metabolomes demonstrated the same trends of alteration between HER2-positive and HER2-negative breast cancer patients (Tables 1, 2). However, ten plasma metabolomes were changed only in HER2-positive breast cancer patients (Table 3). These metabolomes were involved in the metabolism of glycine, phenylalanine, phosphatidic acid, phosphatidylcholine, phosphatidylethanolamine, phosphatidylinositol, and phosphatidylserine (Table 3). Interestingly, six plasma metabolomes were changed only in HER2-negative breast cancer patients (Table 3). These metabolomes were involved in the metabolism of glutamine, tryptophan, and phosphatidylglycerol (Table 3). These findings suggested that the effects of doxorubicin on the alterations of several amino acid and phospholipid metabolism were dependent on the HER2 status.

**Table 3:** List of plasma metabolomes that were significantly altered (FDR < 0.05) only in HER2-positive or HER2-negative breast cancer patients at 2 weeks after completion of doxorubicin treatment

HER2-positive breast cancer patients (total 10 metabolomes)	HER2-negative breast cancer patients (total 6 metabolomes)
• *Glycine*	• **Glutamine**
• **Isoleucine and leucine**	• *Tryptophan*
• **Phenylalanine**	• *Isovalerylcarnitine*
• *Malate*	• *Phosphatidylglycerol (34:1)*
• **Lysophosphatidylinositol (18:1)**	• *Phosphatidylglycerol (36:1)*
• **Phosphatidic acid (34:1)**	• *Phosphatidylglycerol (36:2)*
• **Phosphatidylcholine (36:1)**	
• **Phosphatidylethanolamine (34:1)**	
• **Phosphatidylethanolamine (38:4)**	
• **Phosphatidylserine (40:6)**	

Data are summarized from Tables 1, 2. In fact, Table 1 represents 33 plasma metabolomes that were significantly altered (FDR < 0.05) at 2 weeks after completion of doxorubicin treatment in HER2-positive breast cancer patients, while Table 2 represents 29 plasma metabolomes that were significantly altered (FDR < 0.05) at 2 weeks after completion of doxorubicin treatment in HER2-negative breast cancer patients. According to Table 1, 10 of 33 plasma metabolomes were significantly altered only in HER2-positive breast cancer patients, whereas 23 of 33 plasma metabolomes were also significantly altered in HER2-negative breast cancer patients. According to Table 2, 6 of 29 plasma metabolomes were significantly altered only in HER2-negative breast cancer patients, whereas 23 of 29 plasma metabolomes were also significantly altered in HER2-positive breast cancer patients. Here in Table 3, we showed 10 of 33 plasma metabolomes that were significantly altered only in HER2-positive breast cancer patients and 6 of 29 plasma metabolomes that were significantly altered only in HER2-negative breast cancer patients

**Bold**: Significantly increased when compared to baseline

*Italic*: Significantly decreased when compared to baseline

### Changes in blood metabolomes as severity markers of doxorubicin-induced heart failure

To identify whether the changes in blood metabolomes were associated with the development of doxorubicin-induced heart failure, we calculated the correlations between the absolute changes in altered plasma metabolomes 2 weeks after completion of doxorubicin treatment and the absolute changes in altered cardiac parameters 2 weeks after completion of doxorubicin treatment.

**Fig. 4:**
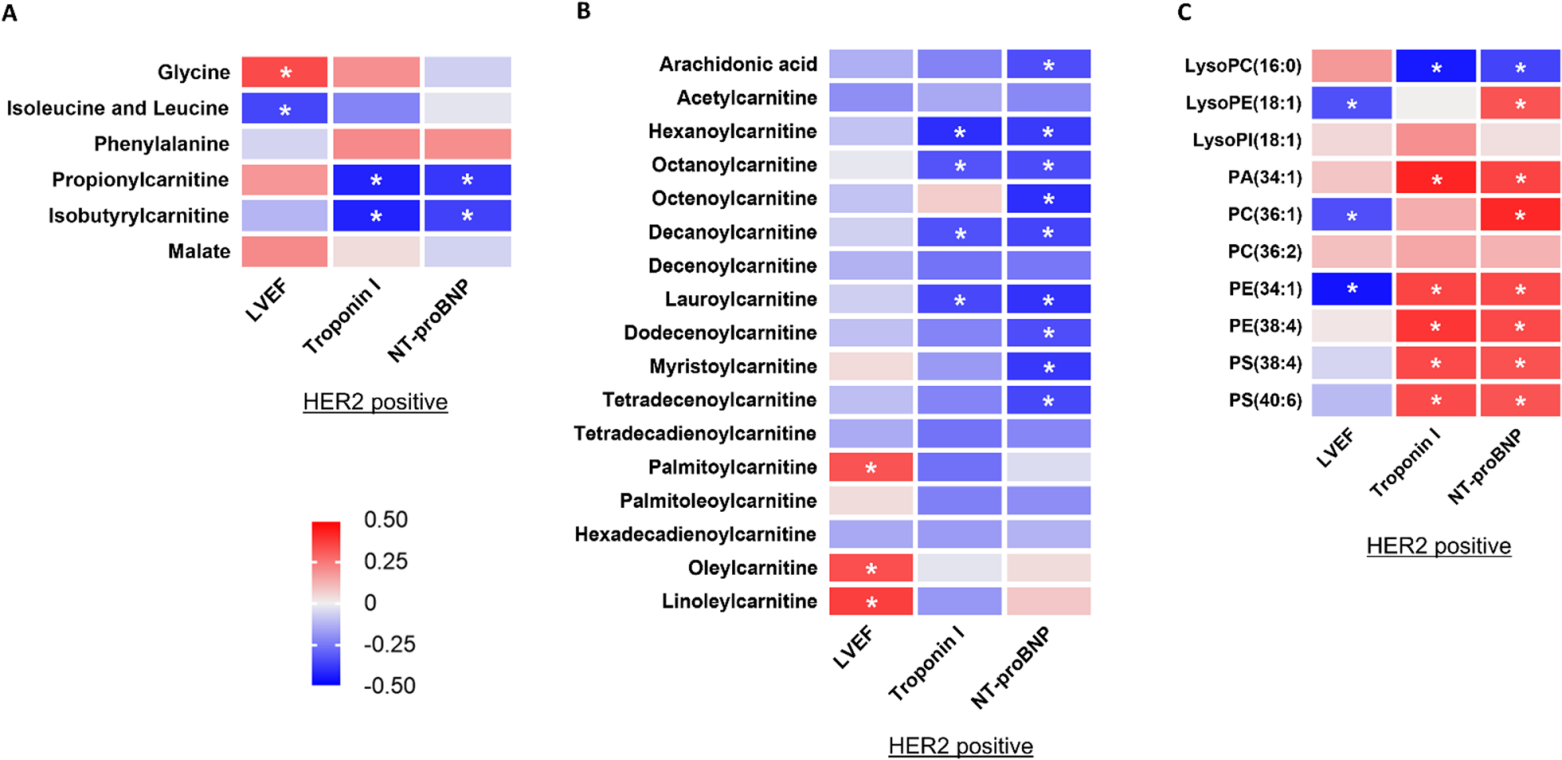
Correlations Between Absolute Changes in Altered Plasma Metabolome Levels 2 Weeks After Completion Of Doxorubicin Treatment Versus Absolute Changes in LVEF And Cardiac Injury Biomarkers 2 Weeks After Completion of Doxorubicin Treatment in HER2-Positive Breast Cancer Patients: (**A**) Metabolomes Involved in Amino Acid Metabolism, (**B**) Metabolomes Involved in Fatty Acid Metabolism, and (**C**) Phospholipids. n = 37. Data are reported as Pearson correlation coefficient value. * *p* < 0.05. LVEF = left ventricular ejection fraction; NT-proBNP = N-terminal pro B-type natriuretic peptide; LysoPC = Lysophosphatidylcholine; LysoPE = Lysophosphatidylethanolamine; LysoPI = Lysophosphatidylinositol; PA = Phosphatidic acid; PC = Phosphatidylcholine; PE = Phosphatidylethanolamine; PS = Phosphatidylserine

In HER2-positive breast cancer patients, the changes in 24 of 33 altered plasma metabolomes 2 weeks after completion of doxorubicin treatment were significantly correlated with the changes in altered cardiac parameters (either LVEF, plasma troponin I or plasma NT-proBNP) 2 weeks after completion of doxorubicin treatment, as detailed in Fig. 4.

**Fig. 5:**
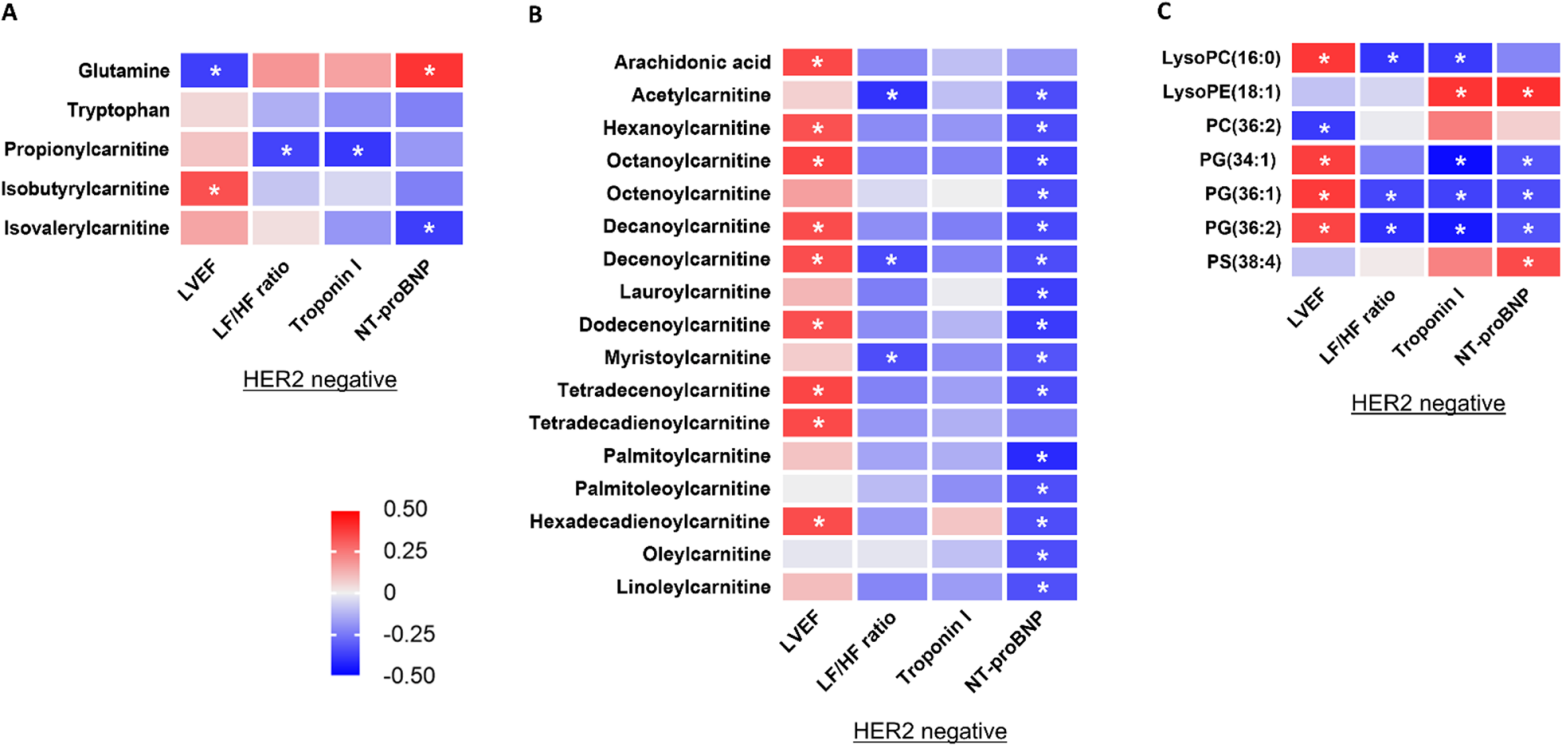
Correlations Between Absolute Changes in Altered Plasma Metabolome Levels 2 Weeks After Completion of Doxorubicin Treatment Versus Absolute Changes in LVEF, LF/HF Ratio, and Cardiac Injury Biomarkers 2 Weeks After Completion of Doxorubicin Treatment in HER2-Negative Breast Cancer Patients: (**A**) Metabolomes Involved in Amino Acid Metabolism, (**B**) Metabolomes Involved in Fatty Acid Metabolism, and (**C**) Phospholipids. n = 37. Data are reported as Pearson correlation coefficient value. * *p* < 0.05. LVEF = Left ventricular ejection fraction; LF/HF ratio = Low frequency-to-high frequency ratio; NT-proBNP = N-terminal pro-B-type natriuretic peptide. LysoPC = Lysophosphatidylcholine; LysoPE = Lysophosphatidylethanolamine; PC = Phosphatidylcholine; PG = Phosphatidylglycerol; PS = Phosphatidylserine

In HER2-negative breast cancer patients, the changes in 28 of 29 altered plasma metabolomes 2 weeks after completion of doxorubicin treatment were significantly correlated with the changes in altered cardiac parameters (either LVEF, LF/HF ratio, plasma troponin I, or plasma NT-proBNP) 2 weeks after completion of doxorubicin treatment, as detailed in Fig. 5. Interestingly, among six plasma metabolomes that were significantly changed only in HER2-negative breast cancer patients, the decreases in only phosphatidylglycerol (36:1) and phosphatidylglycerol (36:2) were significantly correlated with an increase in LF/HF ratio, which was found only in HER2-negative breast cancer patients (Fig. 5).

Among 23 plasma metabolomes that showed the same trends of alteration between HER2-positive and HER2-negative breast cancer patients, the changes in some of these metabolomes exhibited significant correlations with the changes in cardiac parameters only in HER2-positive or only in HER2-negative breast cancer patients, as detailed in Figs. 4, 5. Particularly, the decreases in medium-chain acylcarnitines derived from fatty acids were significantly correlated with a reduction in LVEF only in HER2-negative breast cancer patients (Figs. 4B, 5B). The top five plasma metabolomes that their alterations were significantly correlated with the changes in each cardiac parameter of HER2-positive and HER2-negative breast cancer patients are also listed in Additional file 1: Table S6. All of these results suggested that the changes in blood metabolomes could be severity markers of doxorubicin-induced heart failure, and some of these changes were dependent on HER2 status.

### Cardiac functions and cardiac injury biomarkers three months after completion of doxorubicin treatment in HER2-negative breast cancer patients

**Fig. 6:**
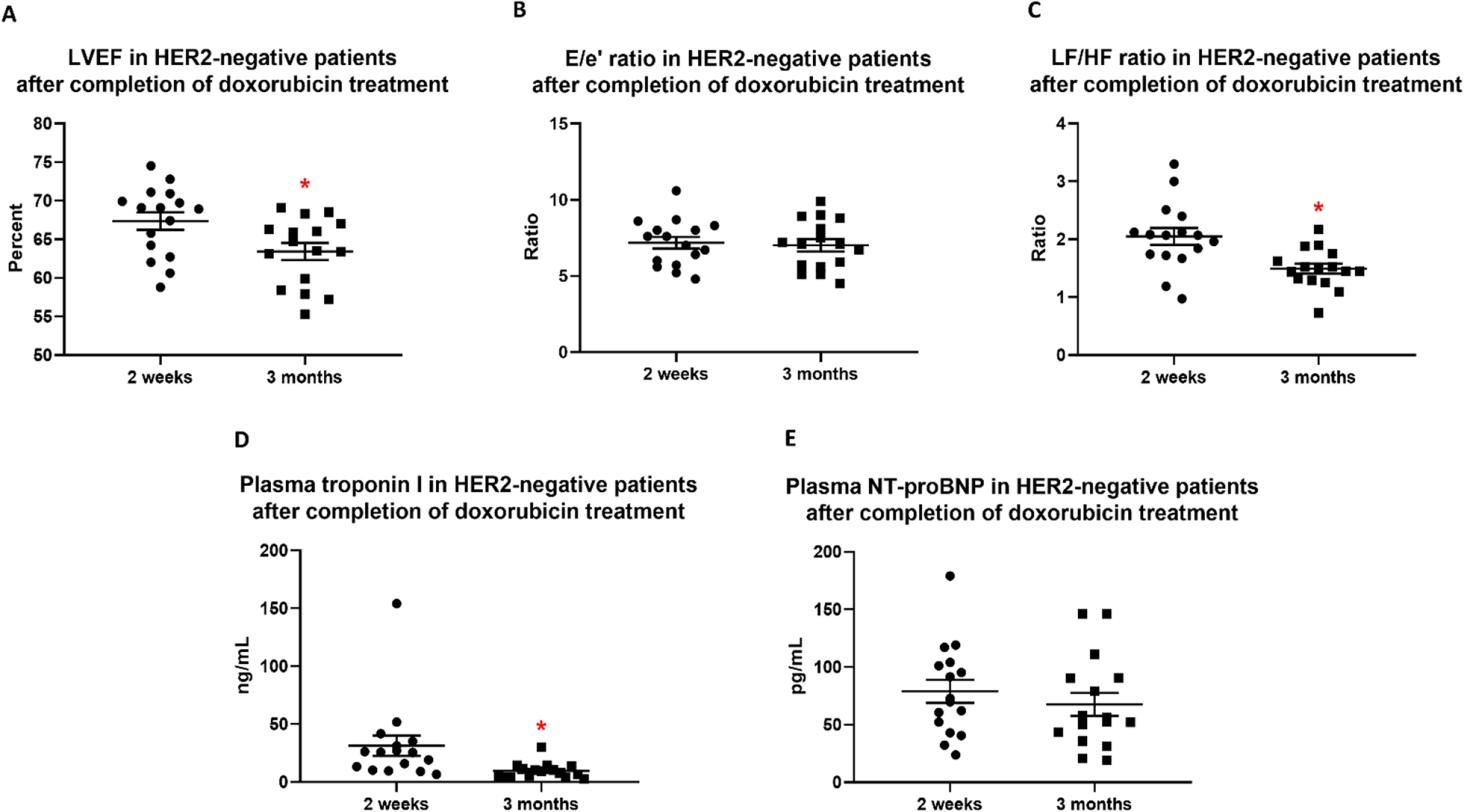
LVEF (**A**), E/e’ Ratio (**B**), LF/HF ratio (**C**), Plasma Troponin I (**D**), and Plasma NT-ProBNP (**E**) in HER2-Negative Breast Cancer Patients 2 Weeks After Completion of Doxorubicin Treatment Versus Three Months After Completion of Doxorubicin Treatment. n = 16. Data are reported as mean ± standard error of the mean (SEM). *p < 0.05 when compared with at 2 weeks after completion of doxorubicin treatment. LVEF = Left ventricular ejection fraction; E/e’ ratio = Early mitral inflow velocity-to-mitral annular early diastolic velocity ratio; LF/HF ratio = Low frequency-to-high frequency ratio; NT-proBNP = N-terminal pro B-type natriuretic peptide

Cardiac functions and cardiac injury biomarkers were also determined three months after completion of doxorubicin treatment. Because trastuzumab treatment might confound the findings, HER2-positive breast cancer patients were excluded from this study timepoint. In addition, 21 of 37 HER2-negative breast cancer patients lost their follow-up at this time point. Therefore, 16 HER2-negative breast cancer patients were included. We found that there was a further decrease in LVEF at 3 months after completion of doxorubicin treatment, as compared with that 2 weeks after completion of doxorubicin treatment (Fig. 6A). E/e’ ratio remained unchanged at this time point (Fig. 6B). Interestingly, the restoration of LF/HF ratio and plasma troponin I level were demonstrated three months after completion of doxorubicin treatment, when compared with those 2 weeks after completion of doxorubicin treatment (Fig. 6C, D). However, plasma NT-proBNP level was not different between these two-time points (Fig. 6E).

### Changes in blood metabolomes as prognostic markers of doxorubicin-induced heart failure

**Fig. 7:**
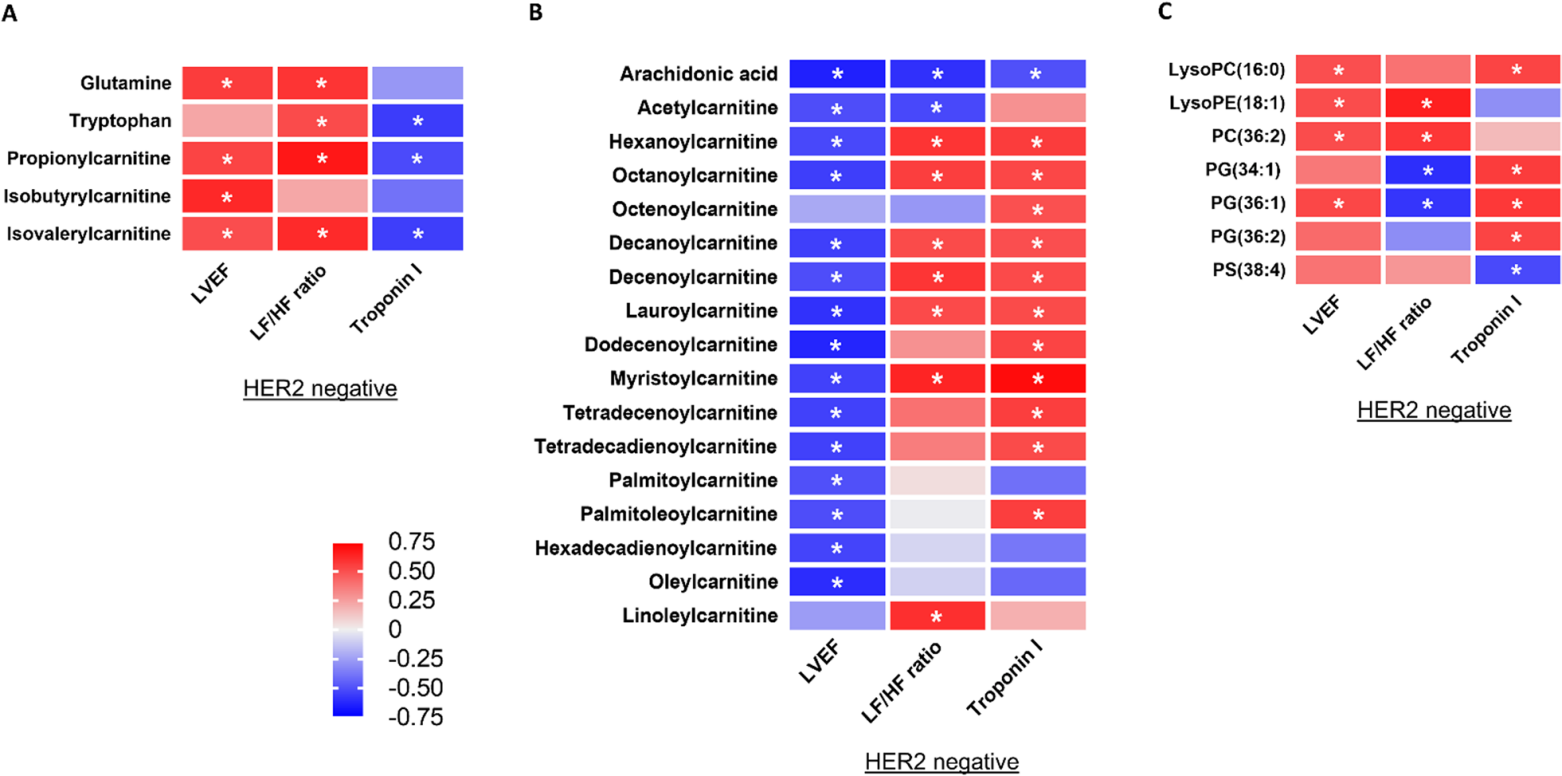
Correlations Between Absolute Changes in Altered Plasma Metabolome Levels 2 Weeks After Completion of Doxorubicin Treatment Versus Absolute Changes in LVEF, LF/HF Ratio, and Plasma Troponin I Three Months After Completion of Doxorubicin Treatment in HER2-Negative Breast Cancer Patients: (**A**) Metabolomes Involved in Amino Acid Metabolism, (**B**) Metabolomes Involved in Fatty Acid Metabolism, and (**C**) Phospholipids. n = 16. Data are reported as Pearson correlation coefficient value. * *p* < 0.05. LVEF = Left ventricular ejection fraction; LF/HF ratio = Low frequency-to-high frequency ratio. LysoPC = Lysophosphatidylcholine; LysoPE = Lysophosphatidylethanolamine; PC = Phosphatidylcholine; PG = Phosphatidylglycerol; PS = Phosphatidylserine

To determine whether the changes in blood metabolomes represented the prognosis of doxorubicin-induced heart failure, we calculated the correlations between the absolute changes in altered plasma metabolomes 2 weeks after completion of doxorubicin treatment versus the absolute changes in altered cardiac parameters three months after completion of doxorubicin treatment. Of 16 HER2-negative breast cancer patients, our results showed that the changes in all 29 altered plasma metabolomes 2 weeks after completion of doxorubicin were significantly correlated with the changes in altered cardiac parameters (either LVEF, LF/HF ratio or plasma troponin I) three months after completion of doxorubicin treatment, as detailed in Fig. 7. These findings suggested that the changes in blood metabolomes could be prognostic markers of doxorubicin-induced heart failure.

## Discussion

It is well-established that doxorubicin exerts an acute cardiotoxic effect via a variety of molecular mechanisms, including oxidative stress, mitochondrial damage, DNA damage, and cell death [34, 35]. All of which occurs within days to months, resulting in myocardial injury and left ventricular (LV) systolic dysfunction [34]. Our findings 2 weeks after completion of doxorubicin treatment supported these pathophysiologic changes, as indicated by an increase in cardiac injury biomarkers along with a decrease in LVEF. Doxorubicin subsequently results in LV remodeling within months to years [34, 35]. These also contribute to LV diastolic dysfunction and worsening of LV systolic dysfunction, i.e., chronic heart failure [34]. Three months after completion of doxorubicin treatment, we observed a further decrease in LVEF, a persistent elevation of plasma NT-proBNP level, and a restoration of plasma troponin I level in our patients. All of these findings indicated doxorubicin-induced chronic heart failure [34, 36, 37]. Nonetheless, LV diastolic dysfunction was not developed in our patients three months after completion of doxorubicin treatment, suggesting that this time point was too early to exhibit doxorubicin-induced LV diastolic dysfunction. This suggestion was supported by previous studies demonstrating that breast cancer patients developed LV diastolic dysfunction 11 months to four years following doxorubicin treatment [38, 39].

Cardiac autonomic dysfunction was previously evidenced in doxorubicin-treated patients at the cumulative dose of 280 mg/m^2^ [40] and 400 mg/m^2^ [41]. According to our findings, the cumulative dose of 240 mg/m^2^ of doxorubicin led to cardiac autonomic dysfunction only in HER2-negative breast cancer patients. Since systemic oxidative stress was found to be strongly associated with the development of cardiac autonomic dysfunction [42, 43], this finding was likely due to an elevation of systemic oxidative stress in these patients 2 weeks after completion of doxorubicin treatment. This result also supported the fact that HER2 expression has been shown to protect against systemic oxidative stress [20–22]. On the other hand, the degree of cardiac systolic dysfunction and injury following doxorubicin treatment were comparable between HER2-positive and HER2-negative breast cancer patients despite the difference of oxidative stress level between these two groups. These findings supported the results from a previous clinical study, in which cardiac systolic function was not correlated with the oxidative stress level [44]. However, future studies are needed to clearly elucidate why doxorubicin-induced cardiac systolic dysfunction and injury was not dependent on HER2 status. Interestingly, cardiac autonomic dysfunction in our HER2-negative breast cancer patients was reversed at 3 months after completion of doxorubicin treatment. This was likely due to an increase in plasma glutamine level that was also observed in these patients 2 weeks after completion of doxorubicin treatment. Glutamine is considered as a potent antioxidant [45]. Hence, increased plasma glutamine levels might have exerted an antioxidative effect against doxorubicin-induced systemic oxidative stress, leading to an improvement of cardiac autonomic function. To support this statement, an additional study measuring antioxidative capacity is required. Additionally, a previous study in mice found that doxorubicin-induced cardiotoxicity was mediated by a recruitment of macrophages in the heart, as indicated by the number of F4/80-positive cells [46]. This consequently resulted in the release of catecholamines and the activation of beta-adrenergic receptor, contributing to cardiac sympathovagal imbalance [46]. Therefore, it was likely that the dynamic alteration of cardiac autonomic function in our HER2-negative breast cancer patients was also mediated by the dynamics changes in macrophage recruitment and catecholamine release. To verify this statement, a future study evaluating F4/80-positive cells and measuring plasma catecholamine levels is necessary.

We found the alterations of branched-chain amino acid-related metabolomes in the plasma of both HER2-positive and HER2-negative breast cancer patients 2 weeks after completion of doxorubicin treatment, which was consistent with our previous study in rats [16]. These results suggested a disruption of branched-chain amino acid oxidation in the hearts of our patients at this time point. Significant correlations were also observed between the changes in some branched-chain amino acid-related metabolomes and a reduction in LVEF in both HER2-positive and HER2-negative breast cancer patients at 2 weeks after completion of doxorubicin. These findings supported the concept that abnormal branched-chain amino acid oxidation is strongly correlated with contractile dysfunction in heart failure [47]. Also consistent with our prior study in rats [13], plasma levels of several fatty acid-derived acylcarnitines were decreased in both HER2-positive and HER2-negative breast cancer patients 2 weeks after completion of doxorubicin treatment. These results suggested that there was a decrease in cardiac fatty acid oxidation following doxorubicin treatment [13]. Importantly, these findings supported the fact that decreased fatty acid oxidation is a major metabolic reprogramming of non-insulin-resistant-induced heart failure [29].

In HER2-positive breast cancer patients, a decrease in plasma glycine level was revealed 2 weeks after completion of doxorubicin treatment. Interestingly, a study in mice observed that glycine administration could protect against doxorubicin-induced cardiotoxicity [48]. Therefore, it was likely that this benefit of glycine administration was mainly due to a restoration of plasma glycine level. An increase in plasma phenylalanine level along with a decrease in plasma malate level of HER2-positive breast cancer patients 2 weeks after completion of doxorubicin treatment indicated an abnormality of phenylalanine metabolism [49]. Since abnormal phenylalanine metabolism has been shown to play a critical role in the development of cardiac cellular senescence [50], cardiac cellular senescence induced by abnormal phenylalanine metabolism could be a potential mechanism mediating doxorubicin-induced heart failure in HER2-positive breast cancer patients. Nevertheless, the interplays between HER2-positive status, glycine metabolism, and phenylalanine metabolism should be further clarified in future studies.

In HER2-negative breast cancer patients, a decrease in plasma tryptophan level was exhibited 2 weeks after completion of doxorubicin treatment. It was discovered that a reduction in plasma tryptophan level indicated an increase in tryptophan breakdown, which was associated with the development of cardiovascular diseases via an increase in systemic inflammation [51]. Hence, doxorubicin-induced cardiotoxicity in HER2-negative breast cancer patients may be potentially mediated by increased tryptophan breakdown. To support this statement, an additional study identifying the relationships between HER2-negative status, tryptophan breakdown, and systemic inflammation should be established. As previously mentioned, glutamine is a potent antioxidant [45]. For this reason, an increase in plasma glutamine levels of HER2-negative breast cancer patients 2 weeks after completion of doxorubicin treatment was likely due to a compensatory mechanism to ameliorate doxorubicin-induced systemic oxidative stress. Importantly, this might have been a major mechanism mediating a restoration of heart rate variability in these patients three months after completion of doxorubicin treatment [42, 43]. Interestingly, a prior study in doxorubicin-treated mice also showed that mitochondrial transplantation activated glutamine metabolism, as indicated by an increase in blood glutamine level [52]. This potentially led to a decrease in cardiac oxidative stress and contributing to an alleviation of heart failure [52]. All of these findings were valuable for the development of mitochondrial transplantation to treat doxorubicin-induced heart failure in clinical practice.

The alteration of plasma phospholipid levels 2 weeks after completion of doxorubicin treatment exhibited different patterns between HER2-positive and HER2-negative breast cancer patients. Since systemic oxidative stress leads to the modification of phospholipids [53–55], the different patterns of plasma phospholipid changes between these two groups of patients were likely due to the different changes in systemic oxidative stress. Interestingly, we found a strong correlation between the decrease in plasma phosphatidylglycerol levels and an increase in LF/HF ratio 2 weeks after completion of doxorubicin treatment in HER2-negative breast cancer patients, suggesting a critical role of phosphatidylglycerol in the regulation of heart rate variability. Therefore, the interplays between phosphatidylglycerol, heart rate variability, and systemic oxidative stress should be elucidated in the future.

Unlike a prior study in serum of mice treated with 36 mg/kg of doxorubicin [56], we did not observe the alterations of alanine, aspartate, isoleucine, glutamate, and methionine levels in plasma of our patients at 2 weeks after completing 240 mg/m^2^ of doxorubicin treatment. These contradictory findings may be due to the different degree of doxorubicin-induced toxicity between the two studies. Interestingly, that prior study demonstrated that dexrazoxane could alter several serum metabolome levels, suggesting that the effect of dexrazoxane on cardioprotection against doxorubicin was mediated by the improvement of various metabolic pathways [56]. In other words, these results also highlighted the roles of blood metabolome levels as non-invasive markers of doxorubicin-induced cardiotoxicity. Another previous study in HER2-positive breast cancer patients revealed the reduction of plasma citrate and isocitrate levels in patients who developed systolic heart failure at three months and six months after completion of doxorubicin and trastuzumab treatment [57]. On the other hand, the alterations of plasma citrate and isocitrate levels were not observed in our HER2-positive breast cancer patients at 2 weeks after completion of doxorubicin treatment. All of these findings suggested that trastuzumab treatment also caused the changes in plasma metabolome levels, and hence the long-term cardiac monitoring in HER2-positive breast cancer patients is essential.

Two weeks after completion of doxorubicin treatment, the changes in several altered plasma metabolomes were found to be correlated with the changes in at least one of the altered cardiac parameters, both in HER2-positive and HER2-negative breast cancer patients. Since our targeted plasma metabolomes are involved in metabolic pathways for fuel utilization of the heart and cardiac mitochondrial structure [29, 31], our findings supported the fact that the disruption of cardiac metabolism in heart failure can be represented by the extensive changes in systemic metabolism [14]. Interestingly, significant correlations between the decreases in medium-chain acylcarnitines derived from fatty acids and a reduction in LVEF at this time point were exhibited only in HER2-negative breast cancer patients. These results suggested that the impact of decreased cardiac fatty acid oxidation on the development of LV systolic dysfunction was more apparent in HER2-negative breast cancer patients. In other words, all of our findings highlighted the roles of changes in blood metabolomes as potential severity markers of doxorubicin-induced heart failure, and some of these changes were dependent on HER2 status. However, molecular mechanisms mediating this HER2-dependent manner should be further determined in the future.

In HER2-negative breast cancer patients, we found that the changes in all 29 altered plasma metabolomes 2 weeks after completion of doxorubicin treatment were correlated with the changes in at least one of the altered cardiac parameters three months after completion of doxorubicin treatment. These results suggested that the changes in blood metabolomes could also be used as potential prognostic markers of doxorubicin-induced heart failure. Interestingly, several amino acids and fatty acid-derived acylcarnitines could be used as prognostic markers, but not severity markers of doxorubicin-induced heart failure in these patients. These findings suggested that the alterations of some amino acid and fatty acid metabolism exert a late effect on the heart in doxorubicin-treated patients. Nonetheless, a future study with a longer follow-up duration should be conducted to identify the roles of changes in blood metabolomes as long-term prognostic markers of doxorubicin-induced cardiotoxicity.

The strength of our study was that here we demonstrated for the potential application of blood metabolomes as non-invasive markers for evaluating the severity and prognosis of doxorubicin-induced cardiotoxicity, both in HER2-positive and HER2-negative breast cancer patients. However, our study had some limitations. First, over half of our HER2-negative breast cancer patients lost their follow-up at three months. This might affect the generalizability of the results and the validity and applicability of the conclusions drawn from the study. For this reason, a future study with a greater number of participants is required. In addition, our study failed to clarify the molecular and cellular mechanisms that mediate the interplays between blood metabolome levels, doxorubicin-induced cardiotoxicity, and HER2 status of the patients. Hence, future investigations on these issues are needed.

## Conclusion

We demonstrated that the changes in blood metabolomes were different between HER2-positive and HER2-negative breast cancer patients. In addition, the changes in some blood metabolomes could potentially be used as severity markers of doxorubicin-induced heart failure and were found to be dependent on the HER2 status. These findings suggested that metabolic interventions for doxorubicin-induced heart failure in breast cancer patients should be considered based on their HER2 status. Our findings also highlighted the roles of changes in blood metabolomes as potential prognostic markers of doxorubicin-induced heart failure. For this reason, early metabolic interventions could be novel strategies to protect against doxorubicin-induced heart failure.

### Supplementary Information

**Additional file 1: Fig. S1.** Study protocol. **Fig. S2.** Absolute changes in LVEF (A), LF/HF ratio (B), plasma troponin I (C), plasma NT-proBNP (D), and cellular oxidative stress in peripheral blood mononuclear cells (E) at 2 weeks after completion of doxorubicin treatment in HER2-positive versus HER2-negative breast cancer patients. **Table S1**. Lists of eighty-five targeted plasma metabolomes and the chromatographic technique for each metabolome. **Table S2.** Patients’ characteristics at baseline. **Table S3:** Baseline plasma metabolome levels in HER2-positive versus HER2-negative breast cancer patients. **Table S4.** Plasma metabolome levels in HER2-positive breast cancer patients at baseline versus at 2 weeks after completion of doxorubicin treatment. **Table S5.** Plasma metabolome levels in HER2-negative breast cancer patients at baseline versus at 2 weeks after completion of doxorubicin treatment. **Table S6.** The top five plasma metabolomes that their alterations were significantly correlated with the changes in each cardiac parameter of HER2-positive and HER2-negative breast cancer patients at 2 weeks after completion of doxorubicin treatment.

## Data Availability

The data that support the findings of this study are available from the corresponding author upon reasonable request.

## Abbreviations

DCF: Dichlorofluorescein
DCFH-DA: Dichloro-dihydro-fluorescein diacetate
DNA: Deoxyribonucleic acid
E: Early mitral inflow velocity
E/e’ ratio: Early mitral inflow velocity-to-mitral annular early diastolic velocity ratio
e’: Mitral annular early diastolic velocity
ECG: Electrocardiogram
FDR: False discovery rate
HER2: Human epidermal growth factor receptor 2
HF: High frequency
HILIC: Hydrophilic interaction liquid chromatography
HPLC: High-performance liquid chromatography
LC: Liquid chromatography
LC/Q-TOF MS: Liquid chromatography coupled with quadrupole time-of-flight mass spectrometry
LF: Low frequency
LF/HF ratio: High frequency-to-low frequency ratio
LV: Left ventricular
LVEF: Left ventricular ejection fraction
LysoPC: Lysophosphatidylcholine
LysoPE: Lysophosphatidylethanolamine
LysoPI: Lysophosphatidylinositol
NCCN: National Comprehensive Cancer Network
NT-proBNP: N-terminal pro-B-type natriuretic peptide
PA: Phosphatidic acid
PC: Phosphatidylcholine
PE: Phosphatidylethanolamine
PG: Phosphatidylglycerol
PS: Phosphatidylserine
RCF: Relative centrifugal force
RPLC: Reversed-phase liquid chromatography
RPM: Revolutions per minute
SEM: Standard error of the mean

## References

[1] Sung H, Ferlay J, Siegel RL, Laversanne M, Soerjomataram I, Jemal A, Bray F (2021). Global cancer statistics 2020: GLOBOCAN estimates of incidence and mortality worldwide for 36 cancers in 185 countries. CA Cancer J Clin.

[2] Lei S, Zheng R, Zhang S, Wang S, Chen R, Sun K, Zeng H, Zhou J, Wei W (2021). Global patterns of breast cancer incidence and mortality: a population-based cancer registry data analysis from 2000 to 2020. Cancer Commun.

[3] Britt KL, Cuzick J, Phillips KA (2020). Key steps for effective breast cancer prevention. Nat Rev Cancer.

[4] DeSantis CE, Ma J, Gaudet MM, Newman LA, Miller KD, Goding Sauer A, Jemal A, Siegel RL (2019). Breast cancer statistics, 2019. CA Cancer J Clin.

[5] Gradishar WJ, Moran MS, Abraham J, Abramson V, Aft R, Agnese D, Allison KH, Anderson B, Burstein HJ, Chew H (2023). NCCN guidelines¬Æ insights: breast cancer, version 4.2023. J Natl Compr Canc Netw.

[6] Peto R, Davies C, Godwin J, Gray R, Pan HC, Clarke M, Cutter D, Darby S, McGale P, Taylor C (2012). Comparisons between different polychemotherapy regimens for early breast cancer: meta-analyses of long-term outcome among 100,000 women in 123 randomised trials. Lancet.

[7] Shah AN, Gradishar WJ (2018). Adjuvant anthracyclines in breast cancer: what is their role?. Oncologist.

[8] Dos Santos AF, Tomé FD, Miguel MP, de Menezes LB, Nagib PRA, Campos EC, Soave DF, Celes MRN (2019). Doxorubicin-induced cardiotoxicity and cardioprotective agents: classic and new players in the game. Curr Pharm Des.

[9] Bansal N, Adams MJ, Ganatra S, Colan SD, Aggarwal S, Steiner R, Amdani S, Lipshultz ER, Lipshultz SE (2019). Strategies to prevent anthracycline-induced cardiotoxicity in cancer survivors. Cardiooncology.

[10] Tahover E, Segal A, Isacson R, Rosengarten O, Grenader T, Gips M, Cherny N, Heching NI, Mesika L, Catane R, Gabizon A (2017). Dexrazoxane added to doxorubicin-based adjuvant chemotherapy of breast cancer: a retrospective cohort study with a comparative analysis of toxicity and survival. Anticancer Drugs.

[11] de Baat EC, Mulder RL, Armenian S, Feijen EA, Grotenhuis H, Hudson MM, Mavinkurve-Groothuis AM, Kremer LC, van Dalen EC (2022). Dexrazoxane for preventing or reducing cardiotoxicity in adults and children with cancer receiving anthracyclines. Cochrane Database Syst Rev.

[12] Sun Z, Zhou D, Yang J, Zhang D (2022). Doxorubicin promotes breast cancer cell migration and invasion via DCAF13. FEBS Open Bio.

[13] Thonusin C, Nawara W, Arinno A, Khuanjing T, Prathumsup N, Ongnok B, Chattipakorn SC, Chattipakorn N (2023). Effects of melatonin on cardiac metabolic reprogramming in doxorubicin-induced heart failure rats: a metabolomics study for potential therapeutic targets. J Pineal Res.

[14] Hunter WG, Kelly JP, McGarrah RW, Kraus WE, Shah SH (2016). Metabolic dysfunction in heart failure: diagnostic, prognostic, and pathophysiologic insights from metabolomic profiling. Curr Heart Fail Rep.

[15] McGarrah RW, Crown SB, Zhang GF, Shah SH, Newgard CB (2018). Cardiovascular metabolomics. Circ Res.

[16] Thonusin C, Nawara W, Khuanjing T, Prathumsup N, Arinno A, Ongnok B, Arunsak B, Sriwichaiin S, Chattipakorn SC, Chattipakorn N (2023). Blood metabolomes as non-invasive biomarkers and targets of metabolic interventions for doxorubicin and trastuzumab-induced cardiotoxicity. Arch Toxicol.

[17] Liao N (2016). HER2-positive breast cancer, how far away from the cure?-on the current situation of anti-HER2 therapy in breast cancer treatment and survival of patients. Chin Clin Oncol.

[18] Dean-Colomb W, Esteva FJ (2008). Her2-positive breast cancer: herceptin and beyond. Eur J Cancer.

[19] Loibl S, Gianni L (2017). HER2-positive breast cancer. Lancet.

[20] Victorino VJ, Campos FC, Herrera AC, Colado Simão AN, Cecchini AL, Panis C, Cecchini R (2014). Overexpression of HER-2/neu protein attenuates the oxidative systemic profile in women diagnosed with breast cancer. Tumour Biol.

[21] Vincent DT, Ibrahim YF, Espey MG, Suzuki YJ (2013). The role of antioxidants in the era of cardio-oncology. Cancer Chemother Pharmacol.

[22] Karihtala P, Kauppila S, Soini Y, Arja Jukkola V (2011). Oxidative stress and counteracting mechanisms in hormone receptor positive, triple-negative and basal-like breast carcinomas. BMC Cancer.

[23] Force T, Krause DS, Van Etten RA (2007). Molecular mechanisms of cardiotoxicity of tyrosine kinase inhibition. Nat Rev Cancer.

[24] Negro A, Brar BK, Lee KF (2004). Essential roles of Her2/erbB2 in cardiac development and function. Recent Prog Horm Res.

[25] Crone SA, Zhao YY, Fan L, Gu Y, Minamisawa S, Liu Y, Peterson KL, Chen J, Kahn R, Condorelli G (2002). ErbB2 is essential in the prevention of dilated cardiomyopathy. Nat Med.

[26] Plana JC, Galderisi M, Barac A, Ewer MS, Ky B, Scherrer-Crosbie M, Ganame J, Sebag IA, Agler DA, Badano LP (2014). Expert consensus for multimodality imaging evaluation of adult patients during and after cancer therapy: a report from the American Society of Echocardiography and the European Association of Cardiovascular Imaging. J Am Soc Echocardiogr.

[27] Electrophysiology TF (1996). Heart rate variability. Standards of measurement, physiological interpretation, and clinical use. Task Force of the European Society of Cardiology and the North American Society of Pacing and Electrophysiology. Eur Heart J.

[28] Khuankaew C, Apaijai N, Sawaddiruk P, Jaiwongkam T, Kerdphoo S, Pongsiriwet S, Tassaneeyakul W, Chattipakorn N, Chattipakorn SC (2018). Effect of coenzyme Q10 on mitochondrial respiratory proteins in trigeminal neuralgia. Free Radic Res.

[29] Kolwicz SC, Purohit S, Tian R (2013). Cardiac metabolism and its interactions with contraction, growth, and survival of cardiomyocytes. Circ Res.

[30] Overmyer KA, Thonusin C, Qi NR, Burant CF, Evans CR (2015). Impact of anesthesia and euthanasia on metabolomics of mammalian tissues: studies in a C57BL/6J mouse model. PLoS ONE.

[31] Hatch GM (2004). Cell biology of cardiac mitochondrial phospholipids. Biochem Cell Biol.

[32] Thonusin C, IglayReger HB, Soni T, Rothberg AE, Burant CF, Evans CR (2017). Evaluation of intensity drift correction strategies using MetaboDrift, a normalization tool for multi-batch metabolomics data. J Chromatogr A.

[33] Pang Z, Chong J, Zhou G, de Lima Morais DA, Chang L, Barrette M, Gauthier C, Jacques P, Li S, Xia J (2021). MetaboAnalyst 5.0: narrowing the gap between raw spectra and functional insights. Nucleic Acids Res.

[34] Robinson EL, Azodi M, Heymans S, Heggermont W (2020). Anthracycline-related heart failure: certain knowledge and open questions: where do we stand with chemotherapyinduced cardiotoxicity?. Curr Heart Fail Rep.

[35] Timm KN, Tyler DJ (2020). The role of AMPK activation for cardioprotection in doxorubicin-induced cardiotoxicity. Cardiovasc Drugs Ther.

[36] Cao Z, Jia Y, Zhu B (1820). BNP and NT-proBNP as diagnostic biomarkers for cardiac dysfunction in both clinical and forensic medicine. Int J Mol Sci.

[37] Park KC, Gaze DC, Collinson PO, Marber MS (2017). Cardiac troponins: from myocardial infarction to chronic disease. Cardiovasc Res.

[38] Upshaw JN, Finkelman B, Hubbard RA, Smith AM, Narayan HK, Arndt L, Domchek S, DeMichele A, Fox K, Shah P (2020). Comprehensive assessment of changes in left ventricular diastolic function with contemporary breast cancer therapy. JACC Cardiovasc Imag.

[39] Serrano JM, González I, Del Castillo S, Muñiz J, Morales LJ, Moreno F, Jiménez R, Cristóbal C, Graupner C, Talavera P (2015). Diastolic dysfunction following anthracycline-based chemotherapy in breast cancer patients: incidence and predictors. Oncologist.

[40] Caru M, Corbin D, Périé D, Lemay V, Delfrate J, Drouin S, Bertout L, Krajinovic M, Valérie C, Andelfinger G (2019). Doxorubicin treatments induce significant changes on the cardiac autonomic nervous system in childhood acute lymphoblastic leukemia long-term survivors. Clin Res Cardiol.

[41] Nousiainen T, Vanninen E, Jantunen E, Remes J, Ritanen E, Vuolteenaho O, Hartikainen J (2001). Neuroendocrine changes during the evolution of doxorubicin-induced left ventricular dysfunction in adult lymphoma patients. Clin Sci (Lond).

[42] Lee CH, Shin HW, Shin DG (2020). Impact of oxidative stress on long-term heart rate variability: linear versus non-linear heart rate dynamics. Heart Lung Circ.

[43] Fadaee SB, Beetham KS, Howden EJ, Stanton T, Isbel NM, Coombes JS (2017). Oxidative stress is associated with decreased heart rate variability in patients with chronic kidney disease. Redox Rep.

[44] Mongirdienė A, Liuizė A, Karčiauskaitė D, Mazgelytė E, Liekis A, Sadauskienė I (2023). Relationship between oxidative stress and left ventricle markers in patients with chronic heart failure. Cells.

[45] Durante W (2019). The emerging role of l-glutamine in cardiovascular health and disease. Nutrients.

[46] Gambardella J, Santulli G, Fiordelisi A, Cerasuolo FA, Wang X, Prevete N, Sommella E, Avvisato R, Buonaiuto A, Altobelli GG (2023). Infiltrating macrophages amplify doxorubicin-induced cardiac damage: role of catecholamines. Cell Mol Life Sci.

[47] Karwi QG, Lopaschuk GD (2023). Branched-chain amino acid metabolism in the failing heart. Cardiovasc Drugs Ther.

[48] Shosha MI, El-Ablack FZ, Saad EA (2023). Glycine protects against doxorubicin-induced heart toxicity in mice. Amino Acids.

[49] Akram M (2014). Citric acid cycle and role of its intermediates in metabolism. Cell Biochem Biophys.

[50] Czibik G, Mezdari Z, Murat Altintas D, Br√©hat J, Pini M, d'Humi√®res T, Delmont T, Radu C, Breau M, Liang H, et al. Dysregulated phenylalanine catabolism plays a key role in the trajectory of cardiac aging. Circulation 2021; 144: 559–574. 10.1161/CIRCULATIONAHA.121.054204 34162223

[51] Mangge H, Stelzer I, Reininghaus EZ, Weghuber D, Postolache TT, Fuchs D (2014). Disturbed tryptophan metabolism in cardiovascular disease. Curr Med Chem.

[52] Sun X, Chen H, Gao R, Huang Y, Qu Y, Yang H, Wei X, Hu S, Zhang J, Wang P (2023). Mitochondrial transplantation ameliorates doxorubicin-induced cardiac dysfunction via activating glutamine metabolism. iScience.

[53] Koleini N, Nickel BE, Edel AL, Fandrich RR, Ravandi A, Kardami E (2019). Oxidized phospholipids in Doxorubicin-induced cardiotoxicity. Chem Biol Interact.

[54] Pamplona R (2008). Membrane phospholipids, lipoxidative damage and molecular integrity: a causal role in aging and longevity. Biochim Biophys Acta.

[55] Nasri Z, Ahmadi M, Striesow J, Ravandeh M, von Woedtke T, Wende K (2022). Insight into the impact of oxidative stress on the barrier properties of lipid bilayer models. Int J Mol Sci.

[56] QuanJun Y, GenJin Y, LiLi W, YongLong H, Yan H, Jie L, JinLu H, Jin L, Run G, Cheng G (2017). Protective effects of dexrazoxane against doxorubicin-induced cardiotoxicity: a metabolomic study. PLoS ONE.

[57] Asnani A, Shi X, Farrell L, Lall R, Sebag IA, Plana JC, Gerszten RE, Scherrer-Crosbie M (2020). Changes in citric acid cycle and nucleoside metabolism are associated with anthracycline cardiotoxicity in patients with breast cancer. J Cardiovasc Transl Res.

