# Supplementary material for: Changes in blood metabolomes as potential markers for severity and prognosis in doxorubicin-induced cardiotoxicity: a study in HER2-positive and HER2-negative breast cancer patients
Source: J Transl Med. 2024 Apr 29;22:398. doi: 10.1186/s12967-024-05088-9 (PMC11059746; doi:10.1186/s12967-024-05088-9)
Supplement: Supplementary file 1 — Additional file 1: Fig. S1. Study protocol. Fig. S2. Absolute changes in LVEF (A), LF/HF ratio (B), plasma troponin I (C), plasma NT-proBNP (D), and cellular oxidative stress in peripheral blood mononuclear cells (E) at 2 weeks after completion of doxorubicin treatment in HER2-positive versus HER2-negative breast cancer patients. Table S1. Lists of eighty-five targeted plasma metabolomes and the chromatographic technique for each metabolome. Table S2. Patients’ characteristics at baseline. Table S3: Baseline plasma metabolome levels in HER2-positive versus HER2-negative breast cancer patients. Table S4. Plasma metabolome levels in HER2-positive breast cancer patients at baseline versus at 2 weeks after completion of doxorubicin treatment. Table S5. Plasma metabolome levels in HER2-negative breast cancer patients at baseline versus at 2 weeks after completion of doxorubicin treatment. Table S6. The top five plasma metabolomes that their alterations were significantly correlated with the changes in each cardiac parameter of HER2-positive and HER2-negative breast cancer patients at 2 weeks after completion of doxorubicin treatment. [file 12967_2024_5088_MOESM1_ESM.docx]

**Fig. S1: Study protocol**

**
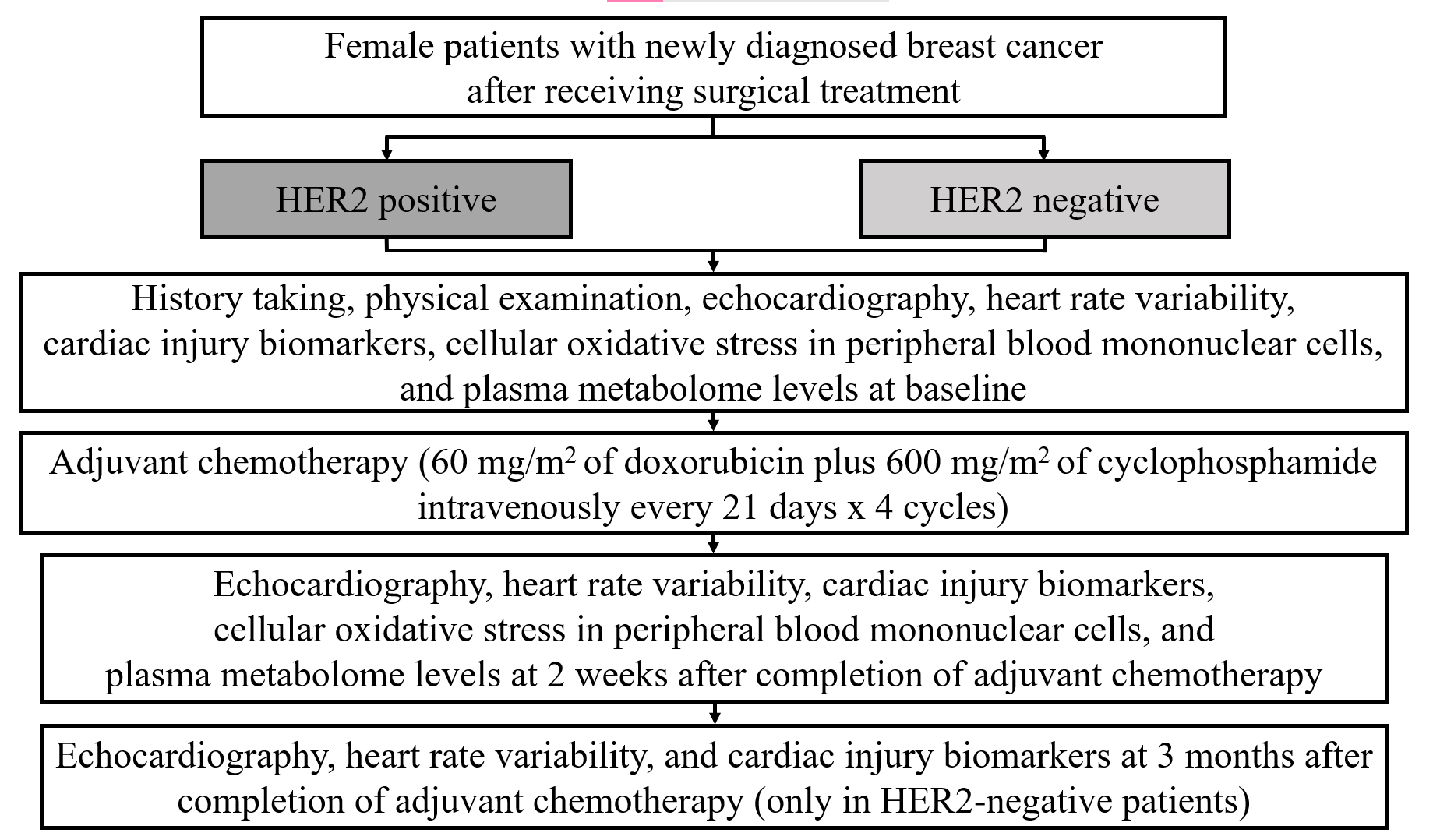
**

**
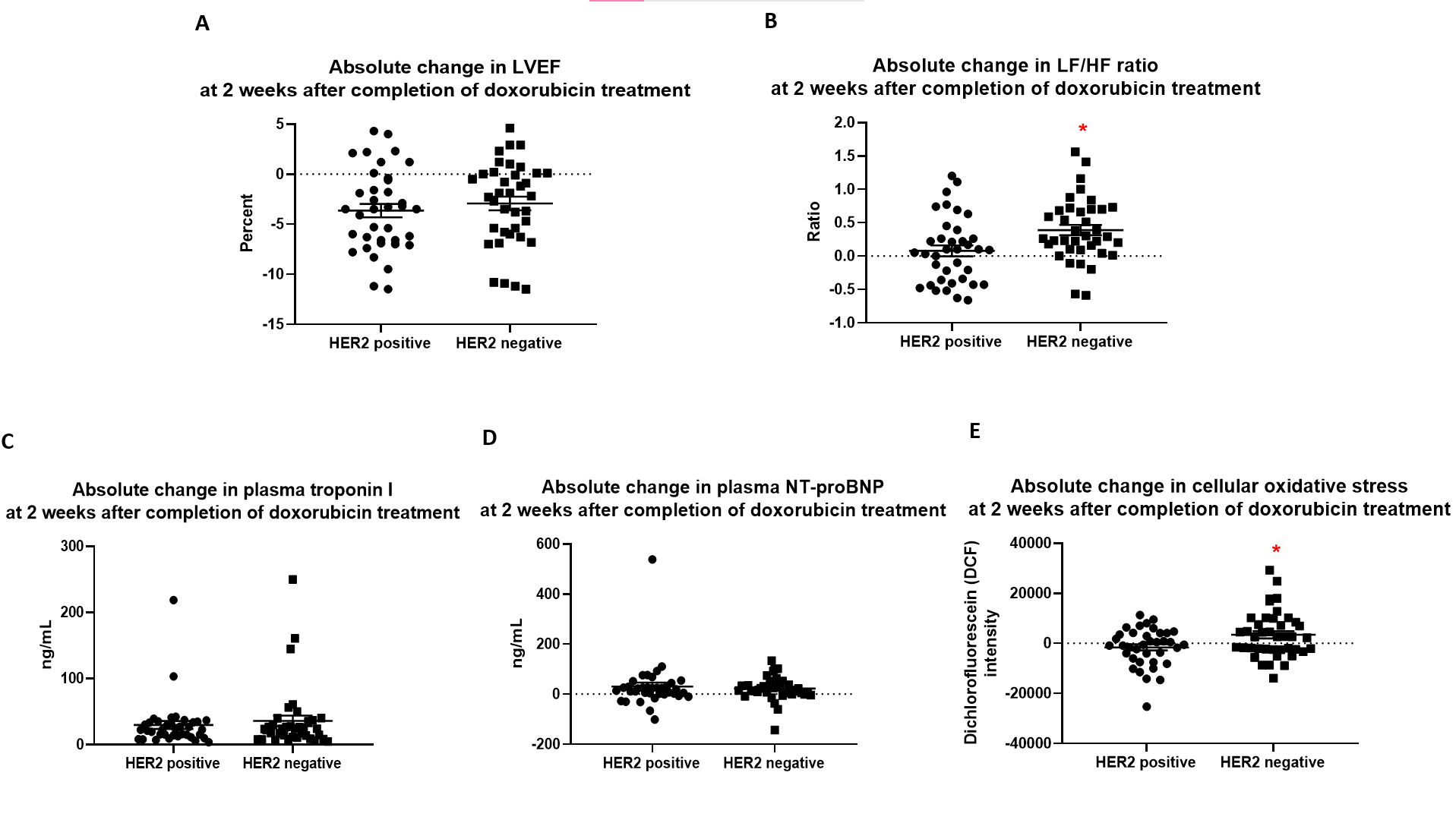
Fig. S2:** **Absolute changes in LVEF (A), LF/HF ratio (B), plasma troponin I (C), plasma NT-proBNP (D), and cellular oxidative stress in peripheral blood mononuclear cells (E) at two weeks after completion of doxorubicin treatment in HER2-positive versus HER2-negative breast cancer patients**

n = 37 per group. Data are reported as mean ± standard error of the mean (SEM). * *p* < 0.05 when compared to HER2-positive breast cancer patients.

LVEF = Left ventricular ejection fraction; LF/HF ratio = Low frequency-to-high frequency ratio; NT-proBNP = N-terminal pro B-type natriuretic peptide; DCF = dichlorofluorescein

**Table S1: Lists of eighty-five targeted plasma metabolomes and the chromatographic technique for each metabolome**

| **Metabolomes** | **Chromatographic technique** |
| --- | --- |
| - Amino acids   - Alanine   - Arginine   - Asparagine   - Aspartate   - Glutamate   - Glutamine   - Glycine   - Histidine   - Isoleucine and Leucine   - Lysine   - Methionine   - Phenylalanine   - Proline   - Threonine   - Tryptophan   - Tyrosine   - Valine | Hydrophilic interaction liquid chromatography negative ion mode |
| - Free fatty acids   - Myristic acid   - Palmitic acid   - Palmitoleic acid   - Stearic acid   - Oleic acid   - Linoleic acid   - Arachidonic acid | Reversed phase liquid chromatography negative ion mode |
| - Acylcarnitines   - Acetylcarnitine   - Propionylcarnitine   - Isobutyrylcarnitine   - Isovalerylcarnitine   - Hexanoylcarnitine   - Octanoylcarnitine   - Octenoylcarnitine   - Decanoylcarnitine   - Decenoylcarnitine   - Lauroylcarnitine   - Dodecenoylcarnitine   - Myristoylcarnitine   - Tetradecenoylcarnitine   - Tetradecadienoylcarnitine   - Palmitoylcarnitine   - Palmitoleoylcarnitine   - Hexadecadienoylcarnitine   - Stearoylcarnitine   - Oleylcarnitine   - Linoleylcarnitine | Reversed phase liquid chromatography positive ion mode |
| - Krebs’ cycle metabolomes   - Citrate and Isocitrate   - Malate   - Succinate | Hydrophilic interaction liquid chromatography negative ion mode |
| - Lactate - Acetoacetate | Hydrophilic interaction liquid chromatography negative ion mode  Hydrophilic interaction liquid chromatography negative ion mode |
| - Phospholipids   - Lysophosphatidylcholine (16:0)   - Lysophosphatidylcholine (18:0)   - Lysophosphatidylcholine (18:1)   - Lysophosphatidylcholine (18:2)   - Lysophosphatidylethanolamine (16:0)   - Lysophosphatidylethanolamine (18:0)   - Lysophosphatidylethanolamine (18:1)   - Lysophosphatidylethanolamine (20:0)   - Lysophosphatidylethanolamine (20:2)   - Lysophosphatidylinositol (18:0)   - Lysophosphatidylinositol (18:1)   - Lysophosphatidylserine(18:0)   - Phosphatidic acid (34:1)   - Phosphatidic acid (36:2)   - Phosphatidylcholine (34:1)   - Phosphatidylcholine (34:2)   - Phosphatidylcholine (36:1)   - Phosphatidylcholine (36:2)   - Phosphatidylcholine (36:4)   - Phosphatidylcholine (38:6)   - Phosphatidylethanolamine (34:1)   - Phosphatidylethanolamine (36:1)   - Phosphatidylethanolamine (36:2)   - Phosphatidylethanolamine (36:3)   - Phosphatidylethanolamine (38:4)   - Phosphatidylethanolamine (38:6)   - Phosphatidylglycerol (34:1)   - Phosphatidylglycerol (36:1)   - Phosphatidylglycerol (36:2)   - Phosphatidylglycerol (38:1)   - Phosphatidylinositol (34:1)   - Phosphatidylinositol (36:1)   - Phosphatidylinositol (36:2)   - Phosphatidylinositol (38:4)   - Phosphatidylserine (38:4)   - Phosphatidylserine (40:6) | Reversed phase liquid chromatography positive ion mode  Reversed phase liquid chromatography positive ion mode  Reversed phase liquid chromatography positive ion mode  Reversed phase liquid chromatography positive ion mode  Reversed phase liquid chromatography negative ion mode  Reversed phase liquid chromatography negative ion mode  Reversed phase liquid chromatography negative ion mode  Reversed phase liquid chromatography negative ion mode  Reversed phase liquid chromatography negative ion mode  Reversed phase liquid chromatography negative ion mode  Reversed phase liquid chromatography negative ion mode  Reversed phase liquid chromatography negative ion mode  Reversed phase liquid chromatography negative ion mode  Reversed phase liquid chromatography negative ion mode  Reversed phase liquid chromatography positive ion mode  Reversed phase liquid chromatography positive ion mode  Reversed phase liquid chromatography positive ion mode  Reversed phase liquid chromatography positive ion mode  Reversed phase liquid chromatography positive ion mode  Reversed phase liquid chromatography positive ion mode  Reversed phase liquid chromatography positive ion mode  Reversed phase liquid chromatography positive ion mode  Reversed phase liquid chromatography positive ion mode  Reversed phase liquid chromatography positive ion mode  Reversed phase liquid chromatography negative ion mode  Reversed phase liquid chromatography negative ion mode  Reversed phase liquid chromatography negative ion mode  Reversed phase liquid chromatography negative ion mode  Reversed phase liquid chromatography negative ion mode  Reversed phase liquid chromatography negative ion mode  Reversed phase liquid chromatography negative ion mode  Reversed phase liquid chromatography negative ion mode  Reversed phase liquid chromatography negative ion mode  Reversed phase liquid chromatography negative ion mode  Reversed phase liquid chromatography negative ion mode  Reversed phase liquid chromatography negative ion mode |

**Table S2: Patients’ characteristics at baseline**

| **Characteristics** | **HER2 positive**  **(n = 37)** | **HER2 negative**  **(n = 37)** | ***p*-value** |
| --- | --- | --- | --- |
| Age (years) | 51.81 ± 1.73 | 49.57 ± 1.81 | 0.37 |
| Body mass index (kg/m^2^) | 23.69 ± 0.59 | 23.76 ± 0.53 | 0.94 |
| Body surface area (m^2^) | 1.59 ± 0.03 | 1.58 ± 0.02 | 0.84 |
| Systolic blood pressure (mmHg) | 129.26 ± 2.08 | 125.19 ± 2.68 | 0.25 |
| Diastolic blood pressure (mmHg) | 74.71 ± 1.35 | 72.92 ± 1.70 | 0.42 |
| Heart rate (beats/min) | 85.80 ± 1.97 | 84.58 ± 1.94 | 0.67 |
| Underlying diseases (n) |  |  |  |
| Hypertension | 6 | 6 | 1.00 |
| Dyslipidemia | 7 | 8 | 0.98 |
| Diabetes mellitus | 0 | 0 | 1.00 |
| Cardiac arrhythmia | 0 | 0 | 1.00 |
| Coronary artery disease | 0 | 0 | 1.00 |
| Valvular heart disease | 0 | 0 | 1.00 |
| Heart failure | 0 | 0 | 1.00 |
| Cerebrovascular disease | 0 | 0 | 1.00 |
| Peripheral artery disease | 0 | 0 | 1.00 |
| Chronic pulmonary disease | 0 | 0 | 1.00 |

n = 37 per group. Data are reported as mean ± standard error of the mean (SEM).

**Table S3: Baseline plasma metabolome levels in HER2-positive versus HER2-negative breast cancer patients**

| **Metabolomes** | **HER2 positive**  **(n = 37)** | **HER2 negative**  **(n = 37)** | **FDR** |
| --- | --- | --- | --- |
| - Amino acids   - Alanine (normalized peak area)   - Arginine (micromole/L)   - Asparagine (normalized peak area)   - Aspartate (micromole/L)   - Glutamate (micromole/L)   - Glutamine (normalized peak area)   - Glycine (micromole/L)   - Histidine (micromole/L)   - Isoleucine and Leucine (micromole/L)   - Lysine (micromole/L)   - Methionine (micromole/L)   - Phenylalanine (micromole/L)   - Proline (micromole/L)   - Threonine (micromole/L)   - Tryptophan (normalized peak area)   - Tyrosine (micromole/L)   - Valine (micromole/L) | 110152.42 ± 6039.52  51.75 ± 3.37  243442.81 ± 7190.03  3.36 ± 0.35  53.65 ± 3.82  8436669.26 ± 201533.41  137.80 ± 12.22  65.17 ± 1.22  93.82 ± 3.21  170.95 ± 4.95  27.37 ± 1.01  55.30 ± 1.81  167.36 ± 9.39  121.74 ± 6.40  2473556.97 ± 82022.44  55.80 ± 1.86  211.58 ± 6.03 | 108241.58 ± 6356.11  52.05 ± 2.97  244033.17 ± 6105.58  3.03 ± 0.33  56.33 ± 4.16  8132458.32 ± 260853.79  130.89 ± 14.45  65.15 ± 1.33  100.69 ± 4.61  172.34 ± 4.85  29.18 ± 1.34  57.61 ± 1.66  174.77 ± 8.57  114.70 ± 4.66  2579633.48 ± 70490.61  57.07 ± 2.18  213.75 ± 8.30 | NS  NS  NS  NS  NS  NS  NS  NS  NS  NS  NS  NS  NS  NS  NS  NS  NS |
| - Branched chain amino acid derived acylcarnitines (nanomole/L) | |  |  |
| - - Propionylcarnitine   - Isobutyrylcarnitine   - Isovalerylcarnitine | 112.77 ± 6.14  148.55 ± 13.29  91.02 ± 5.78 | 120.01 ± 7.16  132.00 ± 8.18  100.51 ± 7.95 | NS  NS  NS |
| - Free fatty acids (micromole/L)   - Myristic acid   - Palmitic acid   - Palmitoleic acid   - Stearic acid   - Oleic acid   - Linoleic acid   - Arachidonic acid | 5.08 ± 0.33  90.90 ± 7.11  18.88 ± 1.57  26.51 ± 2.67  108.03 ± 8.21  59.67 ± 5.30  2.07 ± 0.15 | 4.96 ± 0.38  87.43 ± 6.03  18.29 ± 1.74  25.29 ± 1.67  100.79 ± 7.54  53.52 ± 4.40  1.84 ± 0.12 | NS  NS  NS  NS  NS  NS  NS |
| - Fatty acid-derived acylcarnitines   - Acetylcarnitine (nanomole/L)   - Hexanoylcarnitine (nanomole/L)   - Octanoylcarnitine (nanomole/L)   - Octenoylcarnitine (normalized peak area)   - Decanoylcarnitine (nanomole/L)   - Decenoylcarnitine (normalized peak area)   - Lauroylcarnitine (nanomole/L)   - Dodecenoylcarnitine (normalized peak area)   - Myristoylcarnitine (nanomole/L)   - Tetradecenoylcarnitine (normalized peak area)   - Tetradecadienoylcarnitine (normalized peak area)   - Palmitoylcarnitine (nanomole/L)   - Palmitoleoylcarnitine (normalized peak area)   - Hexadecadienoylcarnitine (normalized peak area)   - Stearoylcarnitine (nanomole/L)   - Oleylcarnitine (normalized peak area)   - Linoleylcarnitine (normalized peak area) | 1030.31 ± 54.62  44.10 ± 3.97  272.27 ± 21.92  2365588.10 ± 211238.14  269.78 ± 21.55  4140566.24 ± 275819.93  60.71 ± 5.07  1570955.03 ± 147257.30  55.72 ± 1.99  2031679.79 ± 212159.98  1278329.93 ± 135272.30  90.21 ± 4.09  1154099.53 ± 81306.01  328454.88 ± 37936.08  13.05 ± 0.53  3670134.61 ± 216533.35  3394534.01 ± 195363.29 | 989.16 ± 52.20  34.73 ± 2.74  236.44 ± 28.03  2113945.39 ± 242657.06  243.87 ± 28.91  3500737.38 ± 288133.27  56.49 ± 4.51  1362416.68 ± 129557.43  52.98 ± 1.35  1717096.15 ± 146327.47  1075549.06 ± 84472.91  87.13 ± 3.14  1037616.79 ± 67499.09  262556.11 ± 21029.23  13.00 ± 0.46  3317232.70 ± 180336.97  3088808.63 ± 207291.51 | NS  NS  NS  NS  NS  NS  NS  NS  NS  NS  NS  NS  NS  NS  NS  NS  NS |
| - Krebs’ cycle metabolomes (micromole/L)   - Citrate and Isocitrate   - Malate   - Succinate | 3.07 ± 0.22  28.75 ± 1.83  66.35 ± 6.68 | 3.32 ± 0.22  26.66 ± 1.65  51.86 ± 5.05 | NS  NS  NS |
| - Lactate (micromole/L) - Acetoacetate (normalized peak area) | 7.56 ± 0.72  55601.27 ± 2202.24 | 8.02 ± 0.79  61902.36 ± 4543.03 | NS  NS |
| - Phospholipids (normalized peak area)   - Lysophosphatidylcholine (16:0)   - Lysophosphatidylcholine (18:0)   - Lysophosphatidylcholine (18:1)   - Lysophosphatidylcholine (18:2)   - Lysophosphatidylethanolamine (16:0)   - Lysophosphatidylethanolamine (18:0)   - Lysophosphatidylethanolamine (18:1)   - Lysophosphatidylethanolamine (20:0)   - Lysophosphatidylethanolamine (20:2)   - Lysophosphatidylinositol (18:0)   - Lysophosphatidylinositol (18:1)   - Lysophosphatidylserine(18:0)   - Phosphatidic acid (34:1)   - Phosphatidic acid (36:2)   - Phosphatidylcholine (34:1)   - Phosphatidylcholine (34:2)   - Phosphatidylcholine (36:1)   - Phosphatidylcholine (36:2)   - Phosphatidylcholine (36:4)   - Phosphatidylcholine (38:6)   - Phosphatidylethanolamine (34:1)   - Phosphatidylethanolamine (36:1)   - Phosphatidylethanolamine (36:2)   - Phosphatidylethanolamine (36:3)   - Phosphatidylethanolamine (38:4)   - Phosphatidylethanolamine (38:6)   - Phosphatidylglycerol (34:1)   - Phosphatidylglycerol (36:1)   - Phosphatidylglycerol (36:2)   - Phosphatidylglycerol (38:1)   - Phosphatidylinositol (34:1)   - Phosphatidylinositol (36:1)   - Phosphatidylinositol (36:2)   - Phosphatidylinositol (38:4)   - Phosphatidylserine (38:4)   - Phosphatidylserine (40:6) | 281502799.77 ± 3698605.74  102746748.09 ± 4021884.41  97423668.53 ± 3375755.18  120879731.41 ± 4163656.94  1171227.98 ± 58694.26  1539190.64 ± 70427.01  976047.04 ± 90639.71  338334.93 ± 15674.96  335039.17 ± 19064.26  119251.48 ± 4490.88  114151.46 ± 8823.12  1484232.85 ± 100754.52  761287.39 ± 80542.27  550610.78 ± 39068.79  218639394.64 ± 2759450.80  251825947.01 ± 2894397.87  146512123.13 ± 4402713.87  241445939.07 ± 3750431.67  186594185.21 ± 2854698.23  104805302.12 ± 2981964.20  1061743.78 ± 40854.33  7528947.07 ± 227912.70  5005154.47 ± 201585.05  2087703.07 ± 272409.51  4404539.87 ± 302844.09  4508026.19 ± 417845.01  6746932.33 ± 536262.65  2119160.07 ± 139694.65  2945402.47 ± 199088.98  57868412.90 ± 2613930.26  1985842.17 ± 193685.36  1279728.54 ± 102988.25  6693027.82 ± 586164.51  43678260.64 ± 3311655.58  388377.30 ± 27104.45  791854.92 ± 32722.94 | 285042932.58 ± 2730123.77  104073129.70 ± 3333198.02  102052632.55 ± 1934043.07  127606342.80 ± 3407945.28  1231389.26 ± 46745.97  1573206.97 ± 44679.08  1094714.40 ± 86111.25  322520.22 ± 15160.41  353858.58 ± 14002.58  112427.75 ± 5563.68  113807.59 ± 7727.79  1337077.92 ± 80973.61  704775.44 ± 72440.59  476323.28 ± 41299.31  222337524.04 ± 2777377.49  252662990.44 ± 2552198.48  147567612.19 ± 4827578.13  240868487.26 ± 3305361.28  187834850.00 ± 2936560.18  103338515.66 ± 3272362.43  1111669.75 ± 51072.37  7855295.91 ± 308907.11  4728317.26 ± 175361.87  2292074.28 ± 308238.98  4137784.11 ± 299236.16  3958455.97 ± 383129.61  6404237.92 ± 567291.13  1974062.13 ± 124111.14  2810982.93 ± 223886.94  55554853.19 ± 2224486.54  1842921.96 ± 169884.17  1182064.16 ± 96209.14  6051779.27 ± 535997.32  43964707.79 ± 4109461.00  370429.66 ± 28814.83  776064.17 ± 38201.26 | NS  NS  NS  NS  NS  NS  NS  NS  NS  NS  NS  NS  NS  NS  NS  NS  NS  NS  NS  NS  NS  NS  NS  NS  NS  NS  NS  NS  NS  NS  NS  NS  NS  NS  NS  NS |

n = 37 per group. Data are reported as mean ± standard error of the mean (SEM).

FDR = False discovery rate; NS = Non-significant (FDR ≥ 0.05)

**Table S4: Plasma metabolome levels in HER2-positive breast cancer patients at baseline versus at two weeks after completion of doxorubicin treatment**

| **Metabolomes** | **Baseline**  **(n = 37)** | **After doxorubicin treatment**  **(n = 37)** | **FDR** |
| --- | --- | --- | --- |
| - Amino acids   - Alanine (normalized peak area)   - Arginine (micromole/L)   - Asparagine (normalized peak area)   - Aspartate (micromole/L)   - Glutamate (micromole/L)   - Glutamine (normalized peak area)   - Glycine (micromole/L)   - Histidine (micromole/L)   - Isoleucine and Leucine (micromole/L)   - Lysine (micromole/L)   - Methionine (micromole/L)   - Phenylalanine (micromole/L)   - Proline (micromole/L)   - Threonine (micromole/L)   - Tryptophan (normalized peak area)   - Tyrosine (micromole/L)   - Valine (micromole/L) | 110152.42 ± 6039.52  51.75 ± 3.37  243442.81 ± 7190.03  3.36 ± 0.35  53.65 ± 3.82  8436669.26 ± 201533.41  137.80 ± 12.22  65.17 ± 1.22  93.82 ± 3.21  170.95 ± 4.95  27.37 ± 1.01  55.30 ± 1.81  167.36 ± 9.39  121.74 ± 6.40  2473556.97 ± 82022.44  55.80 ± 1.86  211.58 ± 6.03 | 106917.54 ± 6392.39  51.30 ± 2.77  253424.09 ± 8278.45  3.51 ± 0.32  62.70 ± 3.41  8845149.11 ± 214965.11  94.78 ± 6.65  60.55 ± 1.81  107.77 ± 3.82  158.39 ± 5.89  27.63 ± 1.02  61.00 ± 1.98  188.14 ± 10.43  110.66 ± 5.00  2202174.19 ± 82022.44  59.58 ± 1.81  219.09 ± 6.80 | NS  NS  NS  NS  NS  NS  0.031  NS  0.030  NS  NS  0.017  NS  NS  NS  NS  NS |
| - Branched chain amino acid derived acylcarnitines (nanomole/L) | |  |  |
| - - Propionylcarnitine   - Isobutyrylcarnitine   - Isovalerylcarnitine | 112.77 ± 6.14  148.55 ± 13.29  91.02 ± 5.78 | 91.61 ± 4.03  92.47 ± 4.61  88.44 ± 6.76 | 0.027  3.22 x 10^-5^  NS |
| - Free fatty acids (micromole/L)   - Myristic acid   - Palmitic acid   - Palmitoleic acid   - Stearic acid   - Oleic acid   - Linoleic acid   - Arachidonic acid | 5.08 ± 0.33  90.90 ± 7.11  18.88 ± 1.57  26.51 ± 2.67  108.03 ± 8.21  59.67 ± 5.30  2.07 ± 0.15 | 4.63 ± 0.42  83.40 ± 8.19  16.19 ± 2.21  23.25 ± 2.31  99.06 ± 10.08  54.10 ± 5.96  1.58 ± 0.14 | NS  NS  NS  NS  NS  NS  0.029 |
| - Fatty acid-derived acylcarnitines   - Acetylcarnitine (nanomole/L)   - Hexanoylcarnitine (nanomole/L)   - Octanoylcarnitine (nanomole/L)   - Octenoylcarnitine (normalized peak area)   - Decanoylcarnitine (nanomole/L)   - Decenoylcarnitine (normalized peak area)   - Lauroylcarnitine (nanomole/L)   - Dodecenoylcarnitine (normalized peak area)   - Myristoylcarnitine (nanomole/L)   - Tetradecenoylcarnitine (normalized peak area)   - Tetradecadienoylcarnitine (normalized peak area)   - Palmitoylcarnitine (nanomole/L)   - Palmitoleoylcarnitine (normalized peak area)   - Hexadecadienoylcarnitine (normalized peak area)   - Stearoylcarnitine (nanomole/L)   - Oleylcarnitine (normalized peak area)   - Linoleylcarnitine (normalized peak area) | 1030.31 ± 54.62  44.10 ± 3.97  272.27 ± 21.92  2365588.10 ± 211238.14  269.78 ± 21.55  4140566.24 ± 275819.93  60.71 ± 5.07  1570955.03 ± 147257.30  55.72 ± 1.99  2031679.79 ± 212159.98  1278329.93 ± 135272.30  90.21 ± 4.09  1154099.53 ± 81306.01  328454.88 ± 37936.08  13.05 ± 0.53  3670134.61 ± 216533.35  3394534.01 ± 195363.29 | 643.90 ± 40.36  22.06 ± 1.34  102.48 ± 10.83  848782.78 ± 61512.31  105.63 ± 13.02  1744072.66 ± 120362.44  32.14 ± 3.10  713378.07 ± 73158.62  47.51 ± 1.42  1094076.37 ± 111976.29  638737.67 ± 58096.89  75.12 ± 3.78  769441.38 ± 61583.07  191378.45 ± 16509.40  12.05 ± 0.50  2585885.16 ± 128635.14  2066021.74 ± 96977.83 | 1.98 x 10^-7^  2.50 x 10^-7^  4.33 x 10^-9^  5.22 x 10^-12^  4.64 x 10^-8^  6.37 x 10^-11^  9.54 x 10^-6^  1.11 x 10^-6^  1.33 x 10^-3^  1.05 x 10^-4^  1.29 x 10^-3^  0.015  3.52 x 10^-6^  1.05 x 10^-4^  NS  1.64 x 10^-4^  5.64 x 10^-8^ |
| - Krebs’ cycle metabolomes (micromole/L)   - Citrate and Isocitrate   - Malate   - Succinate | 3.07 ± 0.22  28.75 ± 1.83  66.35 ± 6.68 | 3.05 ± 0.22  22.66 ± 1.46  56.97 ± 4.21 | NS  0.023  NS |
| - Lactate (micromole/L) - Acetoacetate (normalized peak area) | 7.56 ± 0.72  55601.27 ± 2202.24 | 8.78 ± 0.65  63036.80 ± 2907.20 | NS  NS |
| - Phospholipids (normalized peak area)   - Lysophosphatidylcholine (16:0)   - Lysophosphatidylcholine (18:0)   - Lysophosphatidylcholine (18:1)   - Lysophosphatidylcholine (18:2)   - Lysophosphatidylethanolamine (16:0)   - Lysophosphatidylethanolamine (18:0)   - Lysophosphatidylethanolamine (18:1)   - Lysophosphatidylethanolamine (20:0)   - Lysophosphatidylethanolamine (20:2)   - Lysophosphatidylinositol (18:0)   - Lysophosphatidylinositol (18:1)   - Lysophosphatidylserine(18:0)   - Phosphatidic acid (34:1)   - Phosphatidic acid (36:2)   - Phosphatidylcholine (34:1)   - Phosphatidylcholine (34:2)   - Phosphatidylcholine (36:1)   - Phosphatidylcholine (36:2)   - Phosphatidylcholine (36:4)   - Phosphatidylcholine (38:6)   - Phosphatidylethanolamine (34:1)   - Phosphatidylethanolamine (36:1)   - Phosphatidylethanolamine (36:2)   - Phosphatidylethanolamine (36:3)   - Phosphatidylethanolamine (38:4)   - Phosphatidylethanolamine (38:6)   - Phosphatidylglycerol (34:1)   - Phosphatidylglycerol (36:1)   - Phosphatidylglycerol (36:2)   - Phosphatidylglycerol (38:1)   - Phosphatidylinositol (34:1)   - Phosphatidylinositol (36:1)   - Phosphatidylinositol (36:2)   - Phosphatidylinositol (38:4)   - Phosphatidylserine (38:4)   - Phosphatidylserine (40:6) | 281502799.77 ± 3698605.74  102746748.09 ± 4021884.41  97423668.53 ± 3375755.18  120879731.41 ± 4163656.94  1171227.98 ± 58694.26  1539190.64 ± 70427.01  976047.04 ± 90639.71  338334.93 ± 15674.96  335039.17 ± 19064.26  119251.48 ± 4490.88  114151.46 ± 8823.12  1484232.85 ± 100754.52  761287.39 ± 80542.27  550610.78 ± 39068.79  218639394.64 ± 2759450.80  251825947.01 ± 2894397.87  146512123.13 ± 4402713.87  241445939.07 ± 3750431.67  186594185.21 ± 2854698.23  104805302.12 ± 2981964.20  1061743.78 ± 40854.33  7528947.07 ± 227912.70  5005154.47 ± 201585.05  2087703.07 ± 272409.51  4404539.87 ± 302844.09  4508026.19 ± 417845.01  6746932.33 ± 536262.65  2119160.07 ± 139694.65  2945402.47 ± 199088.98  57868412.90 ± 2613930.26  1985842.17 ± 193685.36  1279728.54 ± 102988.25  6693027.82 ± 586164.51  43678260.64 ± 3311655.58  388377.30 ± 27104.45  791854.92 ± 32722.94 | 265162690.03 ± 3831333.17  90987406.59 ± 3415040.33  95610846.37 ± 2955329.33  131575385.47 ± 3394467.09  1198366.61 ± 44343.67  1565780.06 ± 53356.57  1462488.41 ± 82839.49  297340.99 ± 15246.74  359966.25 ± 14811.45  117904.42 ± 3765.48  137839.59 ± 8321.81  1322243.42 ± 82824.70  1147025.37 ± 105978.00  685892.33 ± 48888.52  224189975.87 ± 3191722.82  252890956.64 ± 3651694.68  163526596.10 ± 3820324.07  247889590.55 ± 3908608.99  183188381.49 ± 3039074.54  99211605.17 ± 2835794.55  1233409.39 ± 46007.73  7191349.94 ± 218863.67  4915382.37 ± 151639.68  1981394.47 ± 210618.09  5676195.34 ± 388157.50  5490523.43 ± 502743.95  6576411.64 ± 432612.35  2053139.25 ± 126486.28  2672066.66 ± 159244.91  60181320.87 ± 2444742.45  2298437.23 ± 164050.13  1478369.95 ± 99943.45  7591556.05 ± 540970.85  55394577.35 ± 3977016.95  568484.87 ± 36424.19  941589.37 ± 39583.73 | 0.014  NS  NS  NS  NS  NS  3.22 x 10^-5^  NS  NS  NS  0.041  NS  2.79 x 10^-3^  NS  NS  NS  0.015  0.046  NS  NS  0.023  NS  NS  NS  0.042  NS  NS  NS  NS  NS  NS  NS  NS  NS  3.37 x 10^-4^  0.023 |

n = 37. Data are reported as mean ± standard error of the mean (SEM).

FDR = False discovery rate; NS = Non-significant (FDR ≥ 0.05)

**Table S5: Plasma metabolome levels in HER2-negative breast cancer patients at baseline versus at two weeks after completion of doxorubicin treatment**

| **Metabolomes** | **Baseline**  **(n = 37)** | **After doxorubicin treatment**  **(n = 37)** | **FDR** |
| --- | --- | --- | --- |
| - Amino acids   - Alanine (normalized peak area)   - Arginine (micromole/L)   - Asparagine (normalized peak area)   - Aspartate (micromole/L)   - Glutamate (micromole/L)   - Glutamine (normalized peak area)   - Glycine (micromole/L)   - Histidine (micromole/L)   - Isoleucine and Leucine (micromole/L)   - Lysine (micromole/L)   - Methionine (micromole/L)   - Phenylalanine (micromole/L)   - Proline (micromole/L)   - Threonine (micromole/L)   - Tryptophan (normalized peak area)   - Tyrosine (micromole/L)   - Valine (micromole/L) | 108241.58 ± 6356.11  52.05 ± 2.97  244033.17 ± 6105.58  3.03 ± 0.33  56.33 ± 4.16  8132458.32 ± 260853.79  130.89 ± 14.45  65.15 ± 1.33  100.69 ± 4.61  172.34 ± 4.85  29.18 ± 1.34  57.61 ± 1.66  174.77 ± 8.57  114.70 ± 4.66  2579633.48 ± 70490.61  57.07 ± 2.18  213.75 ± 8.30 | 101740.96 ± 5977.08  55.53 ± 3.39  258808.88 ± 8273.44  3.43 ± 0.29  58.44 ± 3.43  9052612.57 ± 267387.08  89.80 ± 9.68  62.27 ± 2.55  108.40 ± 5.20  157.90 ± 7.63  28.47 ± 1.70  58.08 ± 2.39  210.56 ± 13.08  120.31 ± 6.36  2061441.00 ± 91160.39  57.72 ± 2.79  216.44 ± 8.69 | NS  NS  NS  NS  NS  0.043  NS  NS  NS  NS  NS  NS  NS  NS  6.05 x 10^-5^  NS  NS |
| - Branched chain amino acid derived acylcarnitines (nanomole/L) | |  |  |
| - - Propionylcarnitine   - Isobutyrylcarnitine   - Isovalerylcarnitine | 120.01 ± 7.16  132.00 ± 8.18  100.51 ± 7.95 | 95.20 ± 5.51  91.04 ± 5.39  72.81 ± 5.44 | 0.023  1.80 x 10^-4^  0.012 |
| - Free fatty acids (micromole/L)   - Myristic acid   - Palmitic acid   - Palmitoleic acid   - Stearic acid   - Oleic acid   - Linoleic acid   - Arachidonic acid | 4.96 ± 0.38  87.43 ± 6.03  18.29 ± 1.74  25.29 ± 1.67  100.79 ± 7.54  53.52 ± 4.40  1.84 ± 0.12 | 4.53 ± 0.43  80.99 ± 9.09  14.43 ± 1.80  26.61 ± 3.55  90.29 ± 10.17  48.46 ± 5.54  1.41 ± 0.14 | NS  NS  NS  NS  NS  NS  0.017 |
| - Fatty acid-derived acylcarnitines   - Acetylcarnitine (nanomole/L)   - Hexanoylcarnitine (nanomole/L)   - Octanoylcarnitine (nanomole/L)   - Octenoylcarnitine (normalized peak area)   - Decanoylcarnitine (nanomole/L)   - Decenoylcarnitine (normalized peak area)   - Lauroylcarnitine (nanomole/L)   - Dodecenoylcarnitine (normalized peak area)   - Myristoylcarnitine (nanomole/L)   - Tetradecenoylcarnitine (normalized peak area)   - Tetradecadienoylcarnitine (normalized peak area)   - Palmitoylcarnitine (nanomole/L)   - Palmitoleoylcarnitine (normalized peak area)   - Hexadecadienoylcarnitine (normalized peak area)   - Stearoylcarnitine (nanomole/L)   - Oleylcarnitine (normalized peak area)   - Linoleylcarnitine (normalized peak area) | 989.16 ± 52.20  34.73 ± 2.74  236.44 ± 28.03  2113945.39 ± 242657.06  243.87 ± 28.91  3500737.38 ± 288133.27  56.49 ± 4.51  1362416.68 ± 129557.43  52.98 ± 1.35  1717096.15 ± 146327.47  1075549.06 ± 84472.91  87.13 ± 3.14  1037616.79 ± 67499.09  262556.11 ± 21029.23  13.00 ± 0.46  3317232.70 ± 180336.97  3088808.63 ± 207291.51 | 678.84 ± 38.45  19.39 ± 1.37  93.12 ± 10.62  975536.66 ± 103282.69  93.71 ± 12.29  1664023.11 ± 122251.98  30.09 ± 2.88  628470.65 ± 67776.48  46.46 ± 1.23  993747.05 ± 98337.15  578485.81 ± 54787.80  73.63 ± 3.81  749699.05 ± 56010.70  177378.74 ± 14657.71  11.75 ± 0.50  2627173.48 ± 156815.41  2351025.30 ± 155929.46 | 2.32 x 10^-5^  1.06 x 10^-5^  1.96 x 10^-7^  1.48 x 10^-5^  1.96 x 10^-7^  9.74 x 10^-8^  1.06 x 10^-5^  7.66 x 10^-6^  3.13 x 10^-3^  3.57 x 10^-4^  2.12 x 10^-5^  0.018  8.01 x 10^-3^  3.78 x 10^-3^  NS  0.017  0.017 |
| - Krebs’ cycle metabolomes (micromole/L)   - Citrate and Isocitrate   - Malate   - Succinate | 3.32 ± 0.22  26.66 ± 1.65  51.86 ± 5.05 | 26.66 ± 1.65  24.43 ± 1.90  49.06 ± 5.21 | NS  NS  NS |
| - Lactate (micromole/L) - Acetoacetate (normalized peak area) | 8.02 ± 0.79  61902.36 ± 4543.03 | 9.12 ± 0.79  63791.63 ± 2491.45 | NS  NS |
| - Phospholipids (normalized peak area)   - Lysophosphatidylcholine (16:0)   - Lysophosphatidylcholine (18:0)   - Lysophosphatidylcholine (18:1)   - Lysophosphatidylcholine (18:2)   - Lysophosphatidylethanolamine (16:0)   - Lysophosphatidylethanolamine (18:0)   - Lysophosphatidylethanolamine (18:1)   - Lysophosphatidylethanolamine (20:0)   - Lysophosphatidylethanolamine (20:2)   - Lysophosphatidylinositol (18:0)   - Lysophosphatidylinositol (18:1)   - Lysophosphatidylserine(18:0)   - Phosphatidic acid (34:1)   - Phosphatidic acid (36:2)   - Phosphatidylcholine (34:1)   - Phosphatidylcholine (34:2)   - Phosphatidylcholine (36:1)   - Phosphatidylcholine (36:2)   - Phosphatidylcholine (36:4)   - Phosphatidylcholine (38:6)   - Phosphatidylethanolamine (34:1)   - Phosphatidylethanolamine (36:1)   - Phosphatidylethanolamine (36:2)   - Phosphatidylethanolamine (36:3)   - Phosphatidylethanolamine (38:4)   - Phosphatidylethanolamine (38:6)   - Phosphatidylglycerol (34:1)   - Phosphatidylglycerol (36:1)   - Phosphatidylglycerol (36:2)   - Phosphatidylglycerol (38:1)   - Phosphatidylinositol (34:1)   - Phosphatidylinositol (36:1)   - Phosphatidylinositol (36:2)   - Phosphatidylinositol (38:4)   - Phosphatidylserine (38:4)   - Phosphatidylserine (40:6) | 285042932.58 ± 2730123.77  104073129.70 ± 3333198.02  102052632.55 ± 1934043.07  127606342.80 ± 3407945.28  1231389.26 ± 46745.97  1573206.97 ± 44679.08  1094714.40 ± 86111.25  322520.22 ± 15160.41  353858.58 ± 14002.58  112427.75 ± 5563.68  113807.59 ± 7727.79  1337077.92 ± 80973.61  704775.44 ± 72440.59  476323.28 ± 41299.31  222337524.04 ± 2777377.49  252662990.44 ± 2552198.48  147567612.19 ± 4827578.13  240868487.26 ± 3305361.28  187834850.00 ± 2936560.18  103338515.66 ± 3272362.43  1111669.75 ± 51072.37  7855295.91 ± 308907.11  4728317.26 ± 175361.87  2292074.28 ± 308238.98  4137784.11 ± 299236.16  3958455.97 ± 383129.61  6404237.92 ± 567291.13  1974062.13 ± 124111.14  2810982.93 ± 223886.94  55554853.19 ± 2224486.54  1842921.96 ± 169884.17  1182064.16 ± 96209.14  6051779.27 ± 535997.32  43964707.79 ± 4109461.00  370429.66 ± 28814.83  776064.17 ± 38201.26 | 264558012.73 ± 4398892.39  92798232.87 ± 4142008.313  98056703.28 ± 3734041.17  140746953.79 ± 4689224.59  1280052.88 ± 83437.48  1661021.03 ± 82558.54  1806523.93 ± 151218.08  296344.13 ± 21523.01  399833.20 ± 22608.74  102796.21 ± 5059.67  118511.20 ± 8635.15  1345812.36 ± 138096.69  1017819.77 ± 129663.01  629130.03 ± 72784.91  222220667.86 ± 2788227.17  260568245.38 ± 3227664.36  158858951.58 ± 4727779.31  256432214.95 ± 3858479.13  187834850.00 ± 2936560.18  93450476.12 ± 3321594.16  1195798.63 ± 53413.86  7366716.47 ± 214358.14  4999058.01 ± 179231.24  2384236.85 ± 265834.76  4852842.10 ± 397620.41  4823668.37 ± 683038.25  5145963.02 ± 545547.17  1706602.06 ± 127443.88  2298012.80 ± 220165.35  57278745.98 ± 2456774.73  1795769.19 ± 214644.10  1293300.92 ± 124872.40  6693524.39 ± 693087.00  44559390.83 ± 4191476.63  573623.97 ± 49338.40  860279.78 ± 45057.57 | 1.14 x 10^-3^  NS  NS  NS  NS  NS  5.20 x 10^-5^  NS  NS  NS  NS  NS  NS  NS  NS  NS  NS  0.017  NS  NS  NS  NS  NS  NS  NS  NS  0.014  0.025  0.010  NS  NS  NS  NS  NS  6.05 x 10^-5^  NS |

n = 37. Data are reported as mean ± standard error of the mean (SEM).

FDR = False discovery rate; NS = Non-significant (FDR ≥ 0.05)

**Table S6: The top five plasma metabolomes that their alterations were significantly correlated with the changes in each cardiac parameter of HER2-positive and HER2-negative breast cancer patients at two weeks after completion of doxorubicin treatment**

| **HER2-positive breast cancer patients** | **HER2-negative breast cancer patients** |
| --- | --- |
| LVEF   1. Phosphatidylethanolamine (34:1) 2. Linoleylcarnitine 3. Isoleucine and leucine 4. Glycine 5. Phosphatidylcholine (36:1) | LVEF   1. Lysophosphatidylcholine (16:0) 2. Phosphatidylglycerol (36:1) 3. Phosphatidylcholine (36:2) 4. Phosphatidylglycerol (34:1) 5. Glutamine |
| Plasma troponin I   1. Lysophosphatidylcholine (16:0) 2. Propionylcarnitine 3. Isobutyrylcarnitine 4. Phosphatidic acid (34:1) 5. Hexanoylcarnitine | Plasma troponin I   1. Phosphatidylglycerol (34:1) 2. Phosphatidylglycerol (36:2) 3. Lysophosphatidylethanolamine (18:1) 4. Propionylcarnitine 5. Lysophosphatidylcholine (16:0) |
| Plasma NT-proBNP   1. Phosphatidylcholine (36:1) 2. Octenoylcarnitine 3. Lauroylcarnitine 4. Propionylcarnitine 5. Myristoylcarnitine | Plasma NT-proBNP   1. Palmitoylcarnitine 2. Lysophosphatidylethanolamine (18:1) 3. Glutamine 4. Dodecenoylcarnitine 5. Isovalerylcarnitine |
|  | LF/HF ratio   1. Phosphatidylglycerol (36:2) 2. Acetylcarnitine 3. Lysophosphatidylcholine (16:0) 4. Propionylcarnitine 5. Phosphatidylglycerol (36:1) |

LVEF = Left ventricular ejection fraction; LF/HF ratio = Low frequency-to-high frequency ratio; NT-proBNP = N-terminal pro B-type natriuretic peptide
